# Gestational Age and Cognitive Development in Childhood

**DOI:** 10.1001/jamanetworkopen.2025.4580

**Published:** 2025-04-14

**Authors:** Samson Nivins, Nelly Padilla, Hedvig Kvanta, Ulrika Ådén

**Affiliations:** 1 Department of Women’s and Children’s Health, Karolinska Institutet, Stockholm, Sweden

## Abstract

**Importance:**

Preterm and early-term births are known risk factors for cognitive impairment, but studies that comprehensively include genetics, prenatal risk, and child-specific factors in high-risk populations are lacking.

**Objective:**

To investigate the long-term cognitive outcomes of children born at various gestational ages, including very preterm (28-31 weeks), moderately preterm (32-33 weeks), late preterm (34-36 weeks), and early term (37-38 weeks), compared with full-term (≥39 weeks), accounting for genetics and other risk factors.

**Design, Setting, and Participants:**

In this prospective, multicenter, longitudinal cross-sectional study, children aged 9 to 10 years were recruited from the Adolescent Brain and Cognitive Development Study between January 1, 2016, and December 31, 2018. Children underwent cognitive assessments using the National Institutes of Health Toolbox, Little Man Task, and Rey Auditory Verbal Learning Test. Polygenic scores for cognitive performance (cogPGS) were generated using results of a genome-wide association study from the genetic variants related to cognitive performance, educational attainment, and mathematical ability. Data analysis was performed from March to June 2024.

**Exposure:**

Preterm (very preterm, moderately preterm, late preterm) and early-term birth status, with full-term birth status as the reference group.

**Main Outcomes and Measures:**

The primary outcome of interest was the composite cognitive score, while secondary outcomes included individual cognitive domain scores. Hierarchical regression models were used to examine associations between gestational age and cognitive outcomes, adjusting for socioeconomic status (SES), cogPGS, prenatal risks, and child-specific factors.

**Results:**

Among 5946 children included in the study (mean [SD] age, 9.9 [0.6] years; 3083 [51.8%] male), 55 (0.9%) were born very preterm, 110 (1.8%) were born moderately preterm, 454 (7.6%) were born late preterm, 261 (4.4%) were born early term, and 5066 (85.2%) were born full term. The cogPGS was positively associated with the composite cognitive score (β = 0.14; 95% CI, 0.12-0.17; *P* < .001) in the overall cohort. Compared with full-term children, those born moderately preterm had lower composite cognitive scores (β = −0.39; 95% CI, −0.55 to −0.22; *P* < .001) and lower scores in vocabulary (β = −0.36; 95% CI, −0.53 to −0.19; *P* < .001), working memory (β = −0.27; 95% CI, −0.45 to −0.09; *P* = .003), episodic memory (β = −0.32; 95% CI, −0.50 to −0.14; *P* < .001), and both short-delay recall (β = −0.36; 95% CI, −0.54 to −0.18; *P* < .001) and long-delay recall (β = −0.29; 95% CI, −0.48 to −0.11; *P* = .002). These associations were independent of SES, cogPGS, and other risk factors. Importantly, the lowest cognitive scores appeared among children born at 32 weeks or less. In contrast, late-preterm and early-term children performed similarly to full-term peers.

**Conclusions and Relevance:**

In this cross-sectional study of children aged 9 to 10 years, moderately preterm birth was associated with long-term cognitive problems independent of SES, genetics, and other risk factors. These findings underscore the need for continued follow-up of all preterm children, with particular focus on those born before 34 weeks’ gestational age, because they may face greater developmental challenges over time.

## Key Points

**Question:**

Is the association between preterm birth and cognitive problems independent of genetic effects?

**Findings:**

In this cross-sectional study involving 5946 children, moderate preterm birth was associated with specific cognitive deficits during childhood independent of genetics, socioeconomic status, and other risk factors, with lower cognition apparent at earlier gestational ages. The association was not influenced by sex.

**Meaning:**

These findings suggest that biological risks such as preterm birth may have long-lasting implications for cognition, independent of genetics and other risk factors.

## Teaser

This cross-sectional study assesses whether preterm and early-term births are associated with lower cognitive scores in children aged 9 to 10 years compared with full-term peers.

## Introduction

Preterm birth (<37 weeks’ gestational age) is a leading cause of child morbidity and mortality,^1,2,3,4^ with 13 million preterm births occurring globally each year.^5,6^ Although advancements in perinatal care have improved survival rates,^7^ these infants remain at higher risk for neurodevelopmental problems, particularly cognitive deficits. Between 24 and 40 weeks of gestation, multiple critical brain developmental processes take place.^8,9^ Premature birth disrupts these processes, leading to dysmaturational events in gray and white matter structures,^10^ which have been strongly associated with cognitive deficits.

Previous studies have found impaired cognitive functions in preterm children,^11,12,13,14,15,16,17,18^ but most studies have focused on extremely preterm (<28 weeks’ gestational age) or very preterm (<32 weeks’ gestational age) births, overlooking moderate to late-preterm (32-36 weeks’ gestational age) births, which make up a substantial portion of preterm births.^19,20^ Recent evidence suggests that children born moderate to late preterm and early term (37-38 weeks’ gestational age) also face elevated risk of cognitive impairments compared with full-term peers (39-40 weeks’ gestational age),^21,22,23,24,25,26,27,28,29,30,31,32^ posing significant public health concerns.

Cognitive abilities are highly heritable, with estimates up to 70%, and this heritability increases from childhood to early adulthood.^33^ Studies examining both heritability and biological risk factors, such as preterm birth, have largely used familial designs,^15,34,35,36^ showing that the association between gestational age and IQ remains independent of shared familial confounders. However, these designs assume siblings share 50% of their genes and inherit the same risk alleles, which is not always the case, and they cannot fully account for specific environmental factors, such as prenatal factors.^37,38,39^ Despite their contributions, familial designs have inherent limitations in fully capturing genetic and environmental influences, highlighting the need for more refined approaches to disentangle these factors in the context of preterm birth and cognitive development. Moreover, most studies have focused on overall IQ rather than specific cognitive domains. Although it is known that preterm children often show delays in cognitive abilities, most perform within the normal range for general cognitive functioning, typically 0.5 to 1.0 SDs below term-born peers.^40,41,42,43^

In the current study, we sought to overcome these limitations by using polygenic scores derived from genome-wide association studies (GWASs) to quantify genetic influences on cognitive outcomes. These scores are aggregating contributions from known genetic variants related to cognitive performance, educational attainment, and mathematical ability based on data from 1.1 million individuals,^44^ explaining 11% to 13% of variance in education attainment and 7% to 10% in cognitive performance. By incorporating polygenic scores into our analysis as covariates, we aimed to control for the genetic variance in cognitive outcomes, allowing for an independent estimation of the association between preterm birth and cognitive outcomes. This approach allows for a more nuanced examination of the association between preterm birth and cognitive outcomes while considering genetic predisposition.

In addition, several nongenetic maternal factors and postnatal factors are known to influence cognitive outcomes in high-risk populations.^13,22,24,45,46,47,48^ However, their independent contributions, alongside genetic influences, remain underexplored, highlighting the need for comprehensive studies that consider both environmental and genetic risks in high-risk populations.

In this cross-sectional study, we investigated whether preterm and early-term births are associated with lower cognitive scores in children aged 9 to 10 years compared with full-term peers, adjusting for genetics, prenatal risks, and child-specific factors. We also investigated the differences in cognitive outcomes across gestational weeks. On the basis of prior studies,^34,35,36^ we hypothesized that biological risks, such as preterm birth, would be associated with long-lasting cognitive outcomes that are independent of genetics and other risk factors, with stronger associations observed for lower gestational age.

## Methods

### Study Participants

The Adolescent Brain and Cognitive Development (ABCD) Study, a prospective cross-sectional study of 11 875 children born between January 1, 2005, and December 31, 2009, to 9987 mothers through 10 801 pregnancies, enrolled children at 9 to 10 years of age (between January 1, 2016, and December 31, 2018). Recruited from 21 data collection sites, the study sample reflects the sociodemographic composition of US children in this age group.^49^ Most participants were enrolled through local schools, whereas some were recruited via community outreach and referrals. Approximately 860 twin pairs were also identified from birth registries. Data for this study were obtained from the National Institute of Mental Health Data Archive, Annual Curated Data Release 5.0. Informed consent or assent was obtained from all participants, and study protocols were approved by the central institutional review board at University of California San Diego. Research methods adhered to relevant guidelines, and data were anonymized. This study followed Strengthening the Reporting of Observational Studies in Epidemiology (STROBE) reporting guideline.

At ages 9 to 10 years, children accompanied by a parent or caregiver completed in-person assessments that included self- or parent-report measures, including neurocognitive assessment, physical health questionnaires, and biological specimen collections.^50,51,52,53,54,55^ Exclusion criteria included extremely preterm birth (<28 weeks’ gestational age), low birth weight (<1200 g), nonproficiency in the English language, neurological problems, or history of seizures. The ABCD Study has not specified reasons for these exclusions or documented the number of children affected. Possible explanations include the distinct developmental trajectories of extremely preterm or very low birth weight children, atypical brain development patterns in those with seizures or neurodevelopmental disorders, and concerns about accurate task completion for participants with limited English language proficiency. To mitigate familial-related factors, we included only 1 child per family at random.

### Neurocognitive Assessment

The neurocognitive battery, designed for completion in 70 minutes,^55^ began with a Snellen vision chart to assess visual acuity, excluding children with legal blindness. The battery includes 10 measures of which 7 are from the National Institutes of Health (NIH) Toolbox: picture vocabulary, oral reading recognition, pattern comparison processing speed, list-sorting working memory, picture sequence memory, flanker inhibitory control and attention, and dimensional change card sort. The ABCD Study team also assessed patients with the Little Man Task and Rey Auditory Verbal Learning Test. All tests were administered on an iPad (Apple, Inc) with one-on-one monitoring by a research assistant. Detailed information on the tests is available in the eMethods in Supplement 1.

### Creating Composite Scores

**Figure 1.**
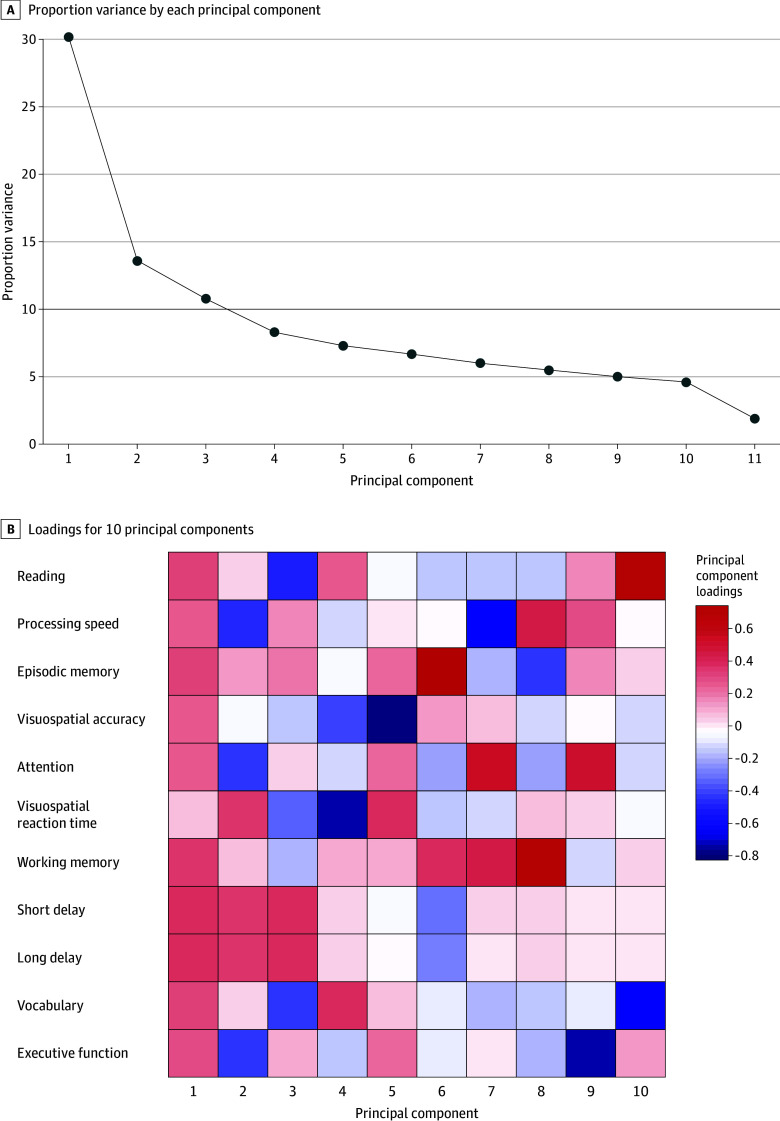
Principal Component Analysis A, The scree plot represents the proportion of variance accounted for by each principal component. B, The heat map displays the loadings for the first 10 principal components identified from the cognitive measures of 5946 children. In this context, red indicates a positive loading, meaning that higher values in the cognitive measures contribute positively to that principal component, whereas blue indicates a negative loading, suggesting that higher values in the cognitive measures are associated with lower contributions to that component.

We aimed to derive both a single composite cognitive score and individual cognitive scores from cognitive assessments studied. Using principal component analysis, we combined the NIH Toolbox, Rey Auditory Verbal Learning Test, and Little Man Task into a single composite cognitive score, extracting the Bartlett factor score from first principal component, accounting for more than 30% of variance (Figure 1). This approach has been used previously to generate a unified composite score.^56^

### Exposures

Gestational age was categorized into very preterm (28-31 weeks), moderate preterm (32-33 weeks), late preterm (34-36 weeks), early term (37-38 weeks), and full term (≥39 weeks [reference]). Gestational age was retrieved from a developmental history questionnaire survey completed by parents or caregivers. Because parents or caregivers only reported gestational age up to 40 weeks, late-term or postterm births could not be studied.

### Covariates and Mediators

Covariates were selected based on prior associations with preterm birth and/or cognitive outcomes.^57,58,59,60,61,62,63,64,65,66,67,68^ These covariates included sociodemographic variables, such as child age, sex assigned at birth, and socioeconomic status (SES), which was calculated based on household income, parental educational level, and neighborhood quality. Maternal characteristics included age, placental problems, hypertensive disorders, diabetes, infections during pregnancy, mode of delivery, alcohol or smoking during pregnancy, and mental health status (depression or anxiety) during pregnancy. Child characteristics included puberty status because it may influence cognitive outcomes. Parents or caregivers reported child race and ethnicity using the ABCD Parent Demographics Survey, selecting from the following categories: Alaska Native, American Indian or Native American, Asian Indian, Black or African American, Chinese, Filipino, Guamanian, Japanese, Korean, Native Hawaiian, Samoan, Other Asian, Other Pacific Islander, Vietnamese, White, other race, refused to answer, or unknown race; and Hispanic, Latino, or Latina ethnicity. Body mass index was calculated as weight in kilograms divided by height in meters squared and subsequently converted to age- and sex-specific percentiles based on the World Health Organization Child Growth Standards for underweight (<5th percentile), normal weight (5th to <85th percentile), overweight (85th to <95th percentile, and obesity (≥95th percentile).

To account for genetic influences on cognitive outcomes, we used polygenic scores for cognitive performance (cogPGS) and the first 20 genetic principal components to account for population stratification within the Add Health European–ancestry subsample. Briefly, we created cogPGS for children using PRSice-2, summing effect sizes of thousands of single-nucleotide variants (SNVs) identified through large GWASs on educational attainment, mathematical ability, and general cognitive ability.^69^ The GWAS data, available through the Social Science Genetics Association Consortium, included effect sizes and *P* values for SNVs. We also incorporated data from a multitrait analysis, combining cognitive performance GWASs with those on educational attainment, highest-level math class completed, and self-reported math ability. This approach was beneficial due to high genetic correlations among traits and large sample sizes involved. To generate polygenic scores, we applied clumping and pruning techniques, removing correlated SNVs within a 250-kilobase window (*r*^2^ > 0.25). We included all SNVs regardless of *P* value, resulting in 5255 SNVs, and normalized the scores (mean [SD], 0 [1]) for fair comparisons across phenotypes.

In addition, neonatal characteristics, such as asphyxia, convulsions, respiratory support, admission to the neonatal intensive care unit, breastfeeding, and jaundice, were considered potential mediators. These factors were treated as mediators because they may lie on the causal pathway between preterm birth and cognitive outcomes, providing insights into the mechanisms by which preterm birth influences cognitive development. Detailed information on the variables is given in eFigures 1 and 2 and the eMethods in Supplement 1.

### Statistical Analysis

Participant characteristics were summarized using means (SDs) for continuous variables and numbers (percentages) for categorical ones. Comparisons of preterm and early-term groups with full-term controls were performed using an unpaired *t* test or Pearson χ^2^ test, as appropriate. The primary outcome was a composite cognitive score, with individual scores as secondary outcomes. The normality of cognitive outcomes was assessed using skewness and histograms with overlaid normal curves. All cognitive variables were normally distributed, with skewness ranging from −2 to 2 (eTable 1 in Supplement 1 and Statistical Analysis Plan in Supplement 2).

To assess associations between gestational age and cognitive outcomes, we used linear mixed-effects models to estimate standardized β coefficients and 95% CIs, using full term as the reference. The models were adjusted for child age, sex, SES, cogPGS, and the first 20 principal components as fixed effects, with scanner sites as random effects (model 1). Analyses were conducted using the lme4 package in R software, version 4.3.1 (R Foundation).

To account for other potential confounders, we applied a stepwise modeling approach. Each subsequent model built on the previous one: model 2 added maternal characteristics to model 1, and model 3 included child characteristics (Statistical Analysis Plan in Supplement 2). We examined correlation between variables (eFigure 3 in Supplement 1) and calculated a generalized variance inflation factor to quantify multicollinearity.^70^ No variance inflation factor values exceeded 2. Model fit was evaluated using quantile-quantile plots for randomly selected cognitive outcomes, which showed a linear pattern, indicating normally distributed residuals. Furthermore, residuals vs fitted values plots were examined and showed no systematic patterns, indicating homoscedasticity. Pairwise Pearson correlation analyses were used to better understand the independent and dependent variables.

The primary outcome was not adjusted for multiple comparisons, with a 2-tailed *P* < .05 considered significant. For secondary outcomes, Bonferroni correction was applied, adjusting the *P* value to .005 (11 tests).

To assess differences in cognitive outcomes across gestational ages, we conducted linear mixed-effects models to estimate β values for each gestational age group (≤32, 33, 34, 35, 36, 37, and 38 weeks), with full term as the reference and adjusted for covariates from model 1. Multiple comparison corrections were not applied, and a 2-tailed *P* < .05 was considered statistically significant.

Given the significant differences observed in the moderately preterm group, we conducted exploratory analyses to investigate the separate associations of sex^14^ with cognitive outcomes by including them as interaction terms in a model adjusted for covariates from model 1. Previous work suggests that preterm births, especially with male sex, increase the risk of intellectual disability.^18^ These analyses were not corrected for multiple comparisons, and a 2-sided *P* < .05 was considered statistically significant. We repeated the moderately preterm group analysis as a sensitivity analysis by restricting the study sample exclusively to children of European ancestry and excluding children with intellectual disability or attention-deficit/hyperactivity disorder (Statistical Analysis Plan in Supplement 2). Mediation analysis was conducted using the mediation package to explore whether neonatal characteristics mediated the association between preterm birth (exposure) and cognitive outcomes (outcome) for significant findings identified in the primary analysis. Mediation analysis was adjusted for all model 1 covariates as described earlier. A 2-sided *P* < .05 was considered statistically significant. All analyses were conducted between March and June 2024 using R, version 4.3.1 (R Foundation).

## Results

### Participant Characteristics

Among the 5946 children included in the study, the mean (SD) age was 9.9 (0.6) years; 3083 (51.8%) were male and 2863 (48.2%) female; 338 (5.7%) were Asian, 92 133 (15.5%) Black, 1150 (19.3%) Hispanic, 24 (0.4%) Native American, 20 (0.3%) Pacific Islander, 3284 (55.2%) White, and 81 (1.4%) other (including responses of other 6 race, refused 34 to answer, and 35 don’t know). A total of 55 (0.9%) were born very preterm, 110 (1.8%) were born moderately preterm, 454 (7.6%) were born late preterm, 261 (4.4%) were born early term, and 5066 (85.2%) were born full term. Mothers of preterm children were more likely than those of full-term children to have undergone cesarean delivery and to have experienced pregnancy-related complications, such as diabetes. However, no differences were found among the groups in terms of maternal smoking, alcohol consumption, drug use, mental health issues, or parental educational level (Table 1; eTable 2 in Supplement 1).

**Table 1.** Maternal and Neonatal Characteristics and Childhood Outcomes of 5946 Children Born Preterm, Early Term, and at Term

Characteristic	No./total No. (%) of children				
	Very preterm (n = 55)	Moderate preterm (n = 110)	Late preterm (n = 454)	Early term (n = 261)	Full term (n = 5066)
Maternal characteristics					
Age at delivery, mean (SD), y	28.0 (6.7)	30.1 (5.9)	30.5 (6.1)^a^	30.8 (5.9)^b^	29.5 (6.2)
Cesarean delivery	32/54 (58.2)^c^	75/109 (68.2)^d^	247/450 (54.4)^a^	122/20 (46.7)^b^	1560/5005 (30.8)
Pregnancy complications^e^	22/54 (40.0)	38/107 (34.5)	127/441 (28.0)	61/255 (23.4)	616/4893 (12.2)
Substance use during pregnancy^f^	3/53 (5.5)	7/107 (6.4)	34/442 (7.5)	21/257 (8.0)	425/491 (8.4)
Mental health status during pregnancy^g^	24/52 (43.6)	48/107 (43.6)	183/429 (40.3)	111/248 (42.5)	2265/4817 (44.3)
Maternal educational level^h^					
Low	15/55 (27.3)	22/110 (20.0)	46/454 (10.1)	30/261 (11.5)	779/5066 (15.4)
High	40/55 (72.7)	88/110 (80.0)	408/454 (89.9)	231/261 (88.5)	4285/5066 (84.6)
Neonatal characteristics					
Gestation length, mean (SD), wk	29.0 (1.6)^c^	32.4 (0.5)^d^	35.3 (0.8)^a^	37.4 (0.5)^b^	40.0 (0.8)
Sex assigned at birth					
Female	22/55 (40.0)	49/110 (44.5)	211/454 (46.5)	114/261 (43.7)	2467/5066(48.7)
Male	33/55 (60.0)	61/110 (55.5)	243/454 (53.5)	147/261 (56.3)	2599/5066 (51.3)
Twin	23/55 (41.8)^c^	60/110 (54.5) ^d^	229/454 (50.4)^a^	65/261 (24.9)^b^	333/5046 (6.6)
Birth weight, mean (SD), kg	1.6 (0.6)^c^	1.8 (0.5)^d^	2.3 (0.5)^a^	2.8 (0.6)^b^	3.2 (0.5)
Neonatal complications^i^	38/53 (69.1)	59/104 (53.6)	156/435 (34.4)	63/250 (24.1)	645/4951 (12.7)
Breastfeeding for at least 6 mo	37/53 (67.3)^c^	67/106 (60.9)^d^	268/444 (59.0)^a^	139/257 (53.3)^b^	2114/4893 (41.7)
Child characteristics					
Age, mean (SD), y	9.9 (0.7)	10.0 (0.6)	10.0 (0.6)	10.0 (0.6)^a^	9.9 (0.6)
Puberty					
Boys					
Early (mean + 1 SD)	5/52 (15.%)	8/104 (13.1)	54/427 (22.2)^a^	24/252 (16.3)	387/4724 (14.9)
Late (mean − 1 SD)	5/52 (15.2)	10/104 (16.4)	32/427 (13.2)	21/252 (14.3)	476/4724 (18.3)
Girls					
Early (mean + 1 SD)	7/52 (31.8)	14/104 (28.6)^d^	32/427 (15.2)	17/252 (14.9)	370/4724 (15.0)
Late (mean − 1 SD)	2/52 (0.9)	6/104 (12.2)	34/427 (16.2)	12/252 (10.5)	396/4724 (16.1)
Body mass index^j^					
Boys					
Overweight	1/55 (3.0)	6/110 (9.8)	22/454 (9.0)	23/261 (15.6)	337/5061 (13.0)
Obesity	15/55 (45.5)	16/110 (26.2)	63/454 (25.9)	37/261 (25.2)	659/5061 (25.4)
Girls					
Overweight	3/55 (13.6)	8/110 (16.3)	27/454 (12.9)	16/261 (14.0)	319/5061 (12.9)
Obesity	8/55 (36.4)	13/110 (26.5)	51/454 (24.3)	26/261 (22.8)	547/5061 (22.2)
Race and ethnicity					
American Indian, Native American, or Alaska Native	1/54 (1.8)	0	16/453 (3.5)	5/259 (1.9)	106/5047 (2.1)
Asian^k^	1/54 (1.8)	2/109 (1.8)	23/453 (5.0)	16/259 (6.1)	296/5047 (5.8)
Black or African American	12/54 (21.8)	19/109 (17.3)	66/453 (14.5)	31/259 (11.9)	793/5047 (15.7)
Hispanic, Latino, or Latina	10/54 (18.2)	28/109 (25.0)	78/453 (17.2)	51/259 (19.5)	983/5047 (19.4)
Pacific Islander^l^	0	1/109 (0.9)	4/453 (0.8)	1/259 (0.3)	1/5047 (0.01)
White	30/54 (54.5)	59/109 (53.6)	263/453 (57.9)	153/259 (58.6)	2779/5047 (54.9)
Other^m^	0	0	3/453 (0.6)	2/259 (0.8)	76/5047 (1.5)
Annual household income, $					
Low (≤$74 999)	34/53 (61.8)	41/100 (37.3)	150/431 (34.8)	90/243 (34.5)	1908/4694 (37.5)
High (≥$75 000)	21/53 (38.2)	69/100 (62.7)	281/431 (65.2)	171/243 (65.5)	3186/4694 (62.5)

^a^ *P* < .05 for comparison between children born late preterm and at full term.

^b^ *P* < .05 for comparison between children born early term and at full term.

^c^ *P* < .05 for comparison between children born very preterm and at full term.

^d^ *P* < .05 for comparison between children born moderate preterm and at full term.

^e^ Diabetes, hypertension disorders, or placenta previa or abruption.

^f^ Any use of alcohol, tobacco, or drugs.

^g^ Presence of depression or emotional or mental problems.

^h^ Low includes middle school or less, some high school, or high school graduate; high includes some college or associate’s, bachelor’s, master’s, or professional degree.

^i^ Jaundice, respiratory support, or convulsions during neonatal period.

^j^ Calculated as weight in kilograms divided by height in meters squared.

^k^ Includes Asian Indian, Chinese, Filipino, Japanese, Korean, Vietnamese, or other Asian.

^l^ Includes Native Hawaiian, Guamanian, Samoan, or other Pacific Islander.

^m^ Includes responses such as other race, refused to answer, and do not know.

As expected, preterm children had lower birth weights and higher rates of postnatal complications, such as need for respiratory support. Girls born moderately preterm reached puberty earlier, whereas boys were more likely to have obesity at 9 to 10 years of age (Table 1; eTable 2 in Supplement 1) than those born full term. Early-term children also had lower birth weights and experienced more postnatal complications compared with full-term peers (Table 1; eTable 2 in Supplement 1).

### Association Between Preterm Birth and Composite Cognitive Outcome

**Figure 2.**
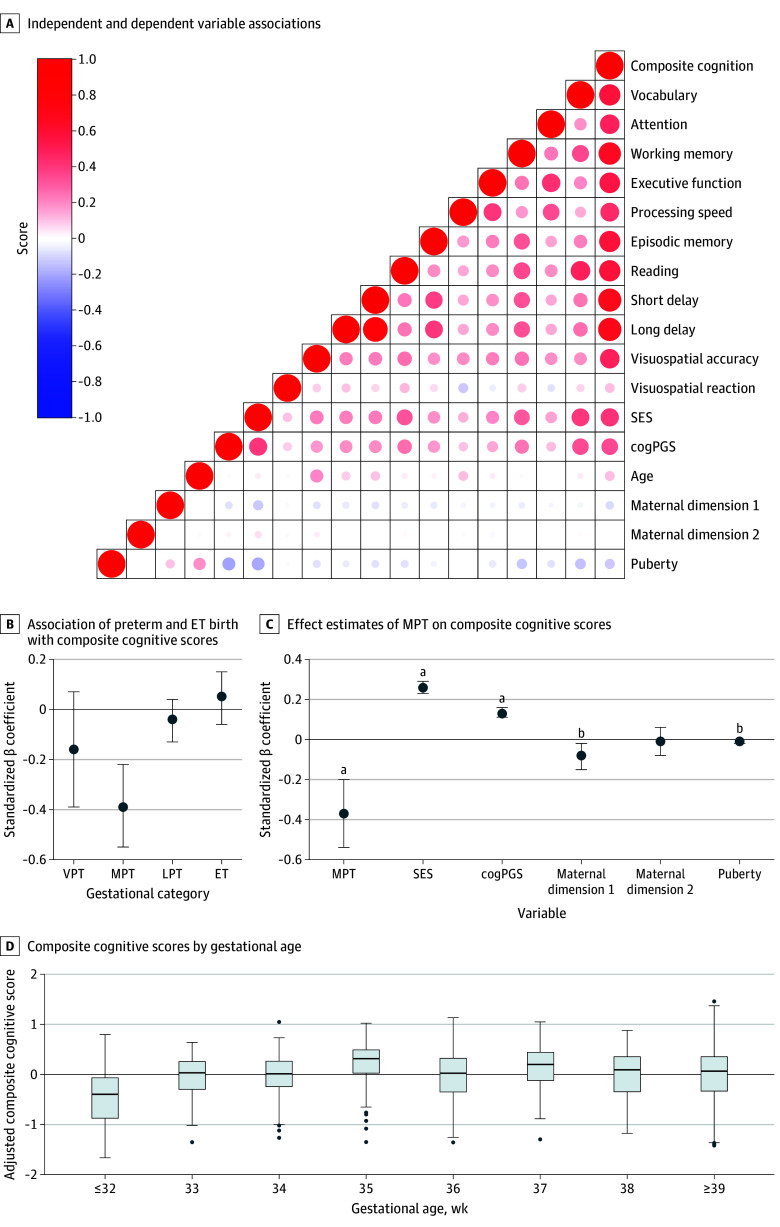
Correlation Analysis A, Correlation matrix showing associations between each independent and dependent variable of interest. B, Association between preterm birth and early-term (ET) birth on composite cognitive scores at 9 to 10 years of age. C, Effect estimates of MPT birth and composite cognitive scores, adjusted for all covariates (model 3). D, Comparative box plot of adjusted composite cognitive scores by weeks of gestational age at 9 to 10 years of age. The model was adjusted for age, sex, socioeconomic status (SES), and polygenic scores for cognitive performance (cogPGS). Maternal dimension 1 and maternal dimension 2 represent the first 2 dimensions derived from the multiple correspondence analysis of maternal characteristics. The ends of the boxes represent the 25th and 75th percentiles; horizontal lines inside the boxes, medians; whiskers, the upper and lower adjacent values; and dots, points that fall beyond the whiskers. Error bars indicate 95% CIs. LPT indicates late preterm; MPT, moderately preterm; VPT, very preterm. ^a^*P* < .001. ^b^*P* < .05.

Compared with full-term peers, children born moderately preterm had lower composite cognitive scores (β = −0.39; 95% CI, −0.55 to −0.22; *P* < .001) at ages 9 to 10 years after adjusting for age, sex, SES, and cogPGS (Table 2 and Figure 2B). When maternal factors were considered, the association was slightly attenuated (β = −0.36; 95% CI, −0.53 to −0.20; *P* < .001). Further adjustment for child factors did not lead to additional changes in the association (β = −0.36; 95% CI, −0.53 to −0.19; *P* < .001) (eTable 3 in Supplement 1; Figure 2C). Given the minimal changes in standardized β estimates across models, model 1 was selected as the primary model for reporting in Table 2 and Figure 2B because it provides a clear assessment of the association while accounting for variables. No significant differences in composite cognitive scores were found for children born very preterm, late preterm, or early term compared with full-term children (Table 2 and Figure 2B).

**Table 2.** Association Between Gestation Length and Cognitive Outcomes in Children Aged 9 to 10 Years Adjusted for Genetic Predisposition (Model 1)^a^

Cognitive outcome	*R* ^2^	VPT (n = 55) vs FT (n = 5066)		MPT (n = 110) vs FT (n = 5066)			LP (n = 454) vs FT (n = 5066)			ET (n = 261) vs FT (n = 5066)	
		β (95% CI)	*P* value	β (95% CI)	*P* value	β (95% CI)		*P* value	β (95% CI)		*P* value
Composite cognitive score	0.25	−0.16 (−0.39 to 0.07)	.17	−0.39 (−0.55 to −0.22)	<.001^b^	−0.04 (−0.13 to 0.04)		.31	0.05 (−0.06 to 0.15)		.42
NIH Toolbox											
Vocabulary	0.24	−0.03 (−0.27 to 0.20)	.10	−0.36 (−0.53 to −0.19)	<.001^b^	−0.02 (−0.11 to 0.06)		.59	0.08 (−0.03 to 0.19)		.14
Attention	0.05	0.01 (−0.26 to 0.27)	.97	−0.08 (−0.27 to 0.10)	.39	0.01 (−0.09 to 0.10)		.93	0.01 (−0.11 to 0.13)		.88
Working memory	0.13	−0.20 (−0.45 to 0.05)	.11	−0.27 (−0.45 to −0.09)	.003^b^	−0.08 (−0.17 to 0.01)		.09	0.003 (−0.11 to 0.12)		.96
Executive function	0.06	−0.12 (−0.38 to 0.14)	.36	−0.07 (−0.25 to 0.12)	.48	0.03 (−0.06 to 0.13)		.47	−0.01 (−0.13 to 0.11)		.87
Processing speed	0.05	−0.14 (−0.40 to 0.12)	.28	−0.09 (−0.28 to 0.10)	.35	−0.04 (−0.13 to 0.06)		.46	0.03 (−0.10 to 0.15)		.67
Episodic memory	0.07	−0.003 (−0.26 to 0.25)	.98	−0.32 (−0.50 to −0.14)	<.001^b^	0.01 (−0.09 to 0.10)		.87	0.10 (−0.02 to 0.22)		.10
Reading	0.15	−0.02 (−0.27 to 0.23)	.87	−0.18 (−0.36 to −0.01)	.04	−0.06 (−0.15 to 0.03)		.21	−0.08 (−0.19 to 0.04)		.19
Rey Auditory Verbal Learning Test											
Short-delay recall	0.10	−0.16 (−0.42 to 0.09)	.20	−0.36 (−0.54 to −0.18)	<.001^b^	−0.002 (−0.10 to 0.09)		.95	0.01 (−0.11 to 0.13)		.92
Long-delay recall	0.10	0.01 (−0.25 to 0.26)	.97	−0.29 (−0.48 to −0.11)	.002^b^	−0.002 (−0.10 to 0.09)		.97	0.05 (−0.07 to 0.17)		.44
Little Man Task											
Visuospatial accuracy	0.13	−0.22 (−0.47 to 0.19)	.08	−0.04 (−0.22 to 0.14)	.67	−0.11 (−0.20 to −0.01)		.03	0.11 (0.00 to 0.23)		.06
Visuospatial reaction time	0.04	−0.31 (−0.58 to −0.05)	.02	−0.20 (−0.39 to −0.01)	.03	0.05 (−0.05 to 0.15)		.34	−0.08 (0.20 to 0.04)		.21

Abbreviations: cogPGS, polygenic scores for cognitive performance; ET, early term (37-38 completed weeks); FT, full term (≥39 completed weeks); LP, late preterm (34-36 completed weeks); MPT, moderate preterm (32-33 completed weeks); NIH, National Institutes of Health; VPT, very preterm.

^a^ The model was adjusted for child age, sex, socioeconomic status, cogPGS, and the first 20 principal components as fixed effects and scanner sites as random effects (model 1).

^b^ *P* values that remain statistically significant after applying multiple comparison corrections.

### Association Between Preterm Birth and Individual Cognitive Outcomes

Children born very preterm exhibited slower visuospatial reaction times (β = −0.31; 95% CI, −0.58 to −0.05; *P* = .02) and a lower, but not statistically significant, visuospatial accuracy (β = −0.22; 95% CI, −0.47 to 0.19; *P* = .08) compared with full-term peers. However, these findings were not significant after multiple comparison corrections (Table 2).

Moderately preterm children had significantly lower scores in vocabulary (β = −0.36; 95% CI, −0.53 to −0.19; *P* < .001), working memory (β = −0.27; 95% CI, −0.45 to −0.09; *P* = .003), episodic memory (β = −0.32; 95% CI, −0.50 to −0.14; *P* < .001), and both short-delay recall (β = −0.36; 95% CI, −0.54 to −0.18; *P* < .001) and long-delay recall (β = −0.29; 95% CI, −0.48 to −0.11; *P* = .002) compared with full-term peers (Table 2). These results persisted even after adjusting for maternal and child characteristics (eTable 3 in Supplement 1). Furthermore, although moderately preterm children also had lower scores in reading skills and slower visuospatial reaction times, these findings did not survive after multiple comparison corrections (Table 2).

Late-preterm children had reduced visuospatial accuracy (β = −0.11; 95% CI, −0.20 to −0.01; *P* = .03) compared with full-term peers, but this association did not survive multiple comparison corrections (Table 2). No significant differences in cognitive scores were found between children born early term and those born full term.

### cogPGS and SES

Both cogPGS and SES were positively associated with the composite cognitive score in the overall cohort. cogPGS had a β of 0.14 (95% CI, 0.12-0.17; *P* < .001), and SES had a β of 0.26 (95% CI, 0.23-0.29; *P* < .001). Both were also positively associated with most individual cognitive scores, with β values ranging from 0.07 to 0.15 for cogPGS and 0.08 to 0.23 for SES (eTable 4 in Supplement 1).

### Association Between Weeks of Gestational Age and Cognitive Outcomes

When we categorized children by gestational age, those born at 32 weeks or earlier (β = −0.35; 95% CI, −0.51 to −0.19; *P* < .001) had lower composite cognitive scores at 9 to 10 years of age after adjusting for age, sex, SES, and cogPGS compared with full-term peers (Figure 2D and Table 3). No significant differences in composite cognitive scores were observed for other gestational age groups. Among the individual cognitive outcomes, children born at 32 weeks or earlier had lower scores in vocabulary, working memory, episodic memory, and both short- and long-delay recall and lower visual reaction times (β ranges from −0.19 to −0.33) compared with full-term peers at 9 to 10 years of age. Moreover, children born at 34 weeks’ gestational age had lower scores in vocabulary (β = −0.19; 95% CI, −0.36 to −0.01) at 9 to 10 years of age (Table 3).

**Table 3.** Association Between Weeks of Gestation and Cognitive Outcomes in Children Aged 9 to 10 Years

Cognitive measure	β (95% CI) by weeks of gestation						
	≤32 (n = 122)	33 (n = 43)	34 (n = 97)	35 (n = 110)	36 (n = 247)	37 (n = 160)	38 (n = 101)
Composite cognitive score	−0.35 (−0.51 to −0.19)^a^	−0.18 (−0.45 to 0.08)^b^	−0.15 (−0.32 to 0.03)^b^	0.04 (−0.12 to 0.21)	−0.04 (−0.15 to 0.070	0.07 (−0.06 to 0.21)	0.001 (−0.17 to 0.21)
NIH Toolbox							
Vocabulary	−0.28 (−0.44 to −0.12)^a^	−0.15 (−0.42 to 0.11)	−0.19 (−0.36 to −0.01)^c^	0.03 (−0.13 to 0.20)	0.02 (−0.10 to 0.13)	0.11 (−0.03 to 0.25)	0.04 (−0.13 to 0.22)
Attention	−0.02 (−0.19 to 0.16)	−0.16 (−0.46 to 0.14)	−0.03 (−0.22 to 0.17)	0.06 (−0.13 to 0.25)	−0.01 (−0.13 to 0.12)	0.03 (−0.12 to 0.19)	−0.03 (−0.22 to 0.17)
Working memory	−0.33 (−0.50 to −0.16)^a^	0.002 (−0.28 to 0.29)	−0.14 (−0.33 to 0.05)^d^	−0.05 (−0.23 to 0.13)	−0.07 (−0.19 to 0.05)	−0.003 (−0.15 to 0.14)	0.01 (−0.17 to 0.20)
Executive function	−0.09 (−0.26 to 0.09)	−0.08 (−0.37 to 0.22)	−0.13 (−0.32 to 0.07)	−0.02 (−0.20 to 0.17)	0.12 (0.01 to 0.25)^d^	−0.02 (−0.17 to 0.13)	0.003 (−0.19 to 0.20)
Processing speed	−0.07 (−0.25 to 0.11)	−0.21 (−0.51 to 0.09)	−0.05 (−0.25 to 0.14)	0.04 (−0.14 to 0.23)	−0.06 (−0.19 to 0.06)	0.04 (−0.12 to 0.19)	0.01 (−0.19 to 0.20)
Episodic memory	−0.21 (−0.38 to −0.03)^c^	−0.23 (−0.53 to 0.06)	0.05 (−0.14 to 0.25)	0.04 (−0.15 to 0.22)	−0.02 (−0.15 to 0.10)	0.05 (−0.10 to 0.20)	0.18 (−0.01 to 0.37)
Reading	−0.19 (−0.36 to −0.02)^c^	0.04 (−0.25 to 0.32)	−0.12 (−0.31 to 0.07)	−0.06 (−0.24 to 0.12)	−0.03 (−0.15 to 0.09)	−0.04 (−0.19 to 0.10)	−0.13 (−0.31 to 0.06)^d^
Rey Auditory Verbal Learning Test							
Short-delay recall	−0.34 (−0.52 to −0.17)^a^	−0.15 (−0.44 to 0.13)	−0.10 (−0.30 to 0.09)	0.18 (0.001 to 0.37)^e^	−0.04 (−0.17 to 0.08)	0.06 (−0.09 to 0.21)	−0.07 (−0.26 to 0.11)
Long-delay recall	−0.24 (−0.42 to −0.07)^b^	−0.05 (−0.34 to 0.24)	−0.04 (−0.23 to 0.15)	0.12 (−0.06 to 0.30)	−0.04 (−0.01 to 0.29)	0.14 (−0.01 to 0.29)^d^	−0.10 (−0.29 to 0.09)
Little Man Task							
Visuospatial accuracy	−0.12 (−0.29 to 0.05)	−0.04 (−0.33 to 0.25)	−0.12 (−0.31 to 0.08)	−0.12 (−0.30 to 0.06)	−0.10 (−0.22 to 0.03)	0.09 (−0.06 to 0.24)	0.16 (−0.03 to 0.34)^d^
Visuospatial reaction time	−0.24 (−0.42 to −0.06)^b^	−0.25 (−0.55 to 0.05)^d^	0.11 (−0.09 to 0.31)	−0.06 (−0.25 to 0.13)	0.07 (−0.06 to 0.20)	−0.11 (−0.26 to 0.05)	−0.03 (−0.23 to 0.16)

Abbreviation: NIH, National Institutes of Health.

^a^ *P* < .001.

^b^ *P* < .01.

^c^ *P* < .05.

^d^ *P* = .10.

^e^ *P* = .05.

### Exploratory, Sensitivity, and Mediation Analyses

Sex did not moderate the association between the gestation and cognitive outcomes in children born moderately preterm (eTable 5 in Supplement 1). However, the results remain inconclusive because the CIs for the interaction terms overlapped with the range observed for group differences, suggesting that the possibility of moderation cannot be ruled out.

Exclusion of children who were not of European ancestry or those with intellectual disability or attention-deficit/hyperactivity disorder did not change the observed association between moderate preterm birth and cognitive impairments during childhood (eTables 6 and 7 in Supplement 1). On a common genetic background, the estimated association of the cogPGS for composite cognitive score increased from 0.14 to 0.18. Neonatal characteristics did not mediate the association between preterm birth and cognitive outcomes after adjusting for covariates (eFigures 4 and 5 in Supplement 1).

## Discussion

Our findings showed that children born moderately preterm, compared with their full-term peers, exhibited lower cognitive scores at 9 to 10 years of age. This association persisted after accounting for prenatal and childhood factors, including SES and polygenic risk, showing that preterm birth was independently associated with long-lasting differences in cognitive development. Neonatal characteristics did not mediate the observed association between moderate preterm birth and cognitive outcomes. In contrast, late-preterm and early-term birth had similar cognitive scores as term-born children. When examining the degree of prematurity in relation to cognitive outcomes, we found that those born at 32 weeks or less demonstrated specific deficits, including language, working memory, and visuospatial skills, compared with full-term peers.

Consistent with previous research using sibling designs,^34,35,36^ we found that lower gestational age was independently associated with lower cognitive scores during mid childhood, regardless of genetic factors. In the current study, we used polygenic scores for cognitive performance to account for the genetic contributions to lower cognitive scores in preterm-born children. Studies have shown positive association between cogPGS and intelligence and school performance.^71,72^ This is one of the first studies, to our knowledge, on preterm birth and cognitive outcomes to use polygenic scores, highlighting the unique contribution of both genetics and preterm birth to cognitive development at 9 to 10 years of age. We found that the standardized β coefficient for cogPGS in relation to composite cognitive scores was 0.14, whereas the coefficient for lower gestational age, particularly in the moderately preterm group, was 2.78 times larger (β = −0.39).

Children born moderately preterm had lower cognitive scores in both composite cognitive measures and specific domains, such as vocabulary and working memory at 9 to 10 years of age, consistent with existing literature despite variations in gestational age definitions and follow-up periods.^29,73^ Furthermore, we explored the association between weeks of gestation and cognitive outcomes in detail. Interestingly, children born at 32 weeks’ gestation or less exhibited lower cognitive scores than full-term peers, unlike those born at 33 weeks. This may be due to increased risks of brain injuries and developmental disruptions before critical brain development milestones.^74^ The cumulative influence of these factors likely contributes to the poor cognitive outcomes in children born before 33 weeks of gestation.

Previous studies have reported atypical language development in very preterm children during the preschool years, with specific deficits in expressive language, receptive language, word retrieval, and short-term auditory memory.^75,76^ These language difficulties often persist into school age.^75^ In contrast, our study found no differences in vocabulary and verbal learning tasks between very preterm children and their full-term peers, although the former exhibited lower scores in working memory and visuospatial skills. One possibility is that these children might have had advantaged familial environments, offering more opportunities for learning and engagement with cognitively stimulating tasks.^77,78^ Another explanation could be that the current study excluded children with severe neurological impairments (eg, cerebral palsy).

Our study found that moderately preterm children had poorer cognitive outcomes than those born very preterm, which appears counterintuitive given the higher risks typically associated with very preterm births. This paradox may be explained by selective survival for which advances in neonatal care mean very preterm children in our study may represent a more resilient subgroup. Furthermore, very preterm children are often identified as high risk and receive targeted early interventions, potentially mitigating cognitive deficits. In contrast, moderately preterm children may not be recognized as needing such support, leaving them more vulnerable to cognitive challenges over time. The smaller sample size in the very preterm group could also limit the ability to detect differences, contributing to this unexpected finding.

Contrary to existing findings,^21,22,23,24,25,26,27,28,29,30,31,32^ late-preterm and early-term children exhibited similar composite and individual cognitive scores compared with their full-term peers at 9 to 10 years of age. Comparison of our results with previous studies is challenging because most studies have focused on cognitive outcomes during the preschool years,^22,23,25,26,28^ with only a few studies extending beyond this period. Moreover, none of the earlier studies have accounted for genetics, which represents a critical methodologic advancement in our research.

### Strengths and Limitations

Our study has several strengths, including a large, prospective, cross-sectional study design and comprehensive neuropsychological assessments, rather than relying solely on IQ. Our participants, born in the late 2000s, reflect current trends in perinatal care and educational curricula. We adjusted for various covariates known to affect gestational age and/or cognitive outcomes, including a genetic factor using polygenic score.

Several limitations should be noted. The polygenic score used to account for genetic contributions explains less than 15% of variation in cognition, indicating that other genetic factors may not have been fully captured, potentially impacting our findings. Maternal, pregnancy, and neonatal information was retrospectively reported by parents and caregivers, which could introduce recall bias. Nonetheless, previous work has demonstrated that maternal recall of birth weight up to 9 years after birth aligns closely with medical records.^79^ We also lacked data on whether gestational length was determined by the mother’s report of her last menstrual period or through ultrasonographic measurement for specific pregnancies, and we did not have details on neonatal morbidities. The smaller subset of children in the very preterm group likely limited our ability to detect differences, reducing the generalizability of our findings to other very preterm populations. In addition, the exclusion of children born extremely preterm, with low birth weight, or with neurologic problems may have introduced selection bias and limited the representativeness of our sample. Finally, although our study is based on a US population, most participants were White, which may limit the generalizability of our findings to populations with different ethnic profiles.

Despite these caveats, our findings have significant implications for health care professionals, educators, and policymakers. Our results suggest that children born before 34 weeks’ gestational age are at higher risk for cognitive problems regardless of genetic or environmental factors. Considering that cognitive impairment is often associated with lower academic achievement and reduced quality of life, our study emphasizes the importance of early screening and targeted interventions for these students, which could greatly benefit their cognitive development.

Future studies should incorporate genetic data related to preterm birth to investigate its influence on cognitive outcomes. Longitudinal studies using genetic data from neonatal stages through older ages are essential for understanding how these genetic factors contribute to cognitive outcomes.

## Conclusions

In this cross-sectional study of children aged 9 to 10 years, moderately preterm birth was associated with a higher risk of long-term cognitive problems, particularly in language, working memory, and episodic memory skills. This association appeared to be independent of genetics and other risk factors, suggesting that biological risks, such as preterm birth, have long-lasting implications for cognition. In contrast, late-preterm or early-term births did not show cognitive problems at 9 to 10 years of age. Given these findings, longer-term follow-up of children born before 34 weeks is warranted, as they may face increased developmental challenges that require more complex cognitive functioning at older ages.

## Notes

Supplement 1. **eMethods.** Supplemental Methods **eTable 1.** Cognitive Outcome Measures of Skewness **eTable 2.** Detailed Maternal and Neonatal Characteristics and Childhood Outcomes of Those Born Preterm, Early Term, and at Term (N = 5946) **eTable 3.** Association Between Moderate Preterm Birth and Cognitive Outcomes in Children Aged 9 to 10 Years (Model 2 and Model 3) **eTable 4.** Association Between ses and cogpgs on Cognitive Outcomes in the Overall Cohort **eTable 5.** Effect of Sex on the Association Between Moderate Preterm Birth and Cognitive Outcomes **eTable 6.** Association Between Moderate Preterm and Cognitive Development at 9 to 10 Years, Excluding Children Who Were Not of European Ancestry **eTable 7.** Association Between Moderate Preterm and Cognitive Development at 9 to 10 Years, Excluding Children With Intellectual Disability or ADHD **eFigure 1.** Multiple Correspondence Analysis (Mca) Factor Map **eFigure 2.** Distributions of Polygenic Scores for Cognitive Performance (PGS) Socioeconomic Status (SES) in the Included Children (N = 5946) **eFigure 3.** Heat map of the Correlation Matrix Between Pairs Covariates **eFigure 4.** Mediation Analysis of First Dimension of Neonatal Factors in Cognitive Outcomes After Moderately Preterm Birth Showing No Mediation **eFigure 5.** Mediation Analysis of Second Dimension of Neonatal Factors in Cognitive Outcomes After Moderately Preterm Birth Showing No Mediation
eReferences

Supplement 2.
Statistical Analysis Plan

Supplement 3.
Data Sharing Statement
