# Supplementary material for: Gestational Age and Cognitive Development in Childhood
Source: JAMA Netw Open. 2025 Apr 14;8(4):e254580. doi: 10.1001/jamanetworkopen.2025.4580 (PMC11997729; doi:10.1001/jamanetworkopen.2025.4580)
Supplement: Supplement 1. — eMethods. Supplemental Methods eTable 1. Cognitive Outcome Measures of Skewness eTable 2. Detailed Maternal and Neonatal Characteristics and Childhood Outcomes of Those Born Preterm, Early Term, and at Term (N = 5946) eTable 3. Association Between Moderate Preterm Birth and Cognitive Outcomes in Children Aged 9 to 10 Years (Model 2 and Model 3) eTable 4. Association Between ses and cogpgs on Cognitive Outcomes in the Overall Cohort eTable 5. Effect of Sex on the Association Between Moderate Preterm Birth and Cognitive Outcomes eTable 6. Association Between Moderate Preterm and Cognitive Development at 9 to 10 Years, Excluding Children Who Were Not of European Ancestry eTable 7. Association Between Moderate Preterm and Cognitive Development at 9 to 10 Years, Excluding Children With Intellectual Disability or ADHD eFigure 1. Multiple Correspondence Analysis (Mca) Factor Map eFigure 2. Distributions of Polygenic Scores for Cognitive Performance (PGS) Socioeconomic Status (SES) in the Included Children (N = 5946) eFigure 3. Heat map of the Correlation Matrix Between Pairs Covariates eFigure 4. Mediation Analysis of First Dimension of Neonatal Factors in Cognitive Outcomes After Moderately Preterm Birth Showing No Mediation eFigure 5. Mediation Analysis of Second Dimension of Neonatal Factors in Cognitive Outcomes After Moderately Preterm Birth Showing No Mediation eReferences [file jamanetwopen-e254580-s001.pdf]

Supplemental Online Content

Nivins S, Padilla N, Kvanta H, Aden U. Gestational age and cognitive development in childhood. *JAMA Netw Open*. 2025;8(4):e254580.
doi:10.1001/jamanetworkopen.2025.4580

eMethods. Supplemental Methods
eTable 1. Cognitive Outcome Measures of Skewness
eTable 2. Detailed Maternal and Neonatal Characteristics and Childhood Outcomes of Those Born Preterm, Early Term, and at Term (N = 5946)
eTable 3. Association Between Moderate Preterm Birth and Cognitive Outcomes in Children Aged 9 to 10 Years (Model 2 and Model 3)
eTable 4. Association Between SES and cogPGS on Cognitive Outcomes in the Overall Cohort
eTable 5. Effect of Sex on the Association Between Moderate Preterm Birth and Cognitive Outcomes
eTable 6. Association Between Moderate Preterm and Cognitive Development at 9 to 10 Years, Excluding Children Who Were Not of European Ancestry
eTable 7. Association Between Moderate Preterm and Cognitive Development at 9 to 10 Years, Excluding Children With Intellectual Disability or ADHD
eFigure 1. Multiple Correspondence Analysis (MCA) Factor Map
eFigure 2. Distributions of Polygenic Scores for Cognitive Performance (PGS) Socioeconomic Status (SES) in the Included Children (N = 5946)
eFigure 3. Heat Map of the Correlation Matrix Between Pairs Covariates
eFigure 4. Mediation Analysis of First Dimension of Neonatal Factors in Cognitive Outcomes After Moderately Preterm Birth Showing No Mediation
eFigure 5. Mediation Analysis of Second Dimension of Neonatal Factors in Cognitive Outcomes After Moderately Preterm Birth Showing no Mediation
eReferences

This supplemental material has been provided by the authors to give readers additional information about their work.

© 2025 Nivins S et al. *JAMA Netw Open*.

eMethods. Supplemental Methods

Cognitive measures

NIH Toolbox cognitive battery

The neurocognitive battery consists of seven different tasks that assess episodic memory, executive function, attention, working memory, processing speed, and language abilities.[1-3] It was normed for individuals aged to 3 and 85 years. The total administration time for the NIH Toolbox Cognitive battery is approximately 35 minutes.

The Toolbox Picture Vocabulary Task measures language skills and verbal intellect.[4,5] The Toolbox Oral Reading Recognition Task evaluates reading ability by asking children to pronounce single words. The Toolbox Pattern Comparison Processing Speed Test assesses rapid visual processing.[6,7] The Toolbox List Sorting Working Memory Test requires children to use working memory to sequence task stimuli based on category membership and perceptual characteristics. The Toolbox Picture Sequence Memory Test, modelled after memory tests, asks children to imitate a sequence of actions using props.[8,9] The Toolbox Flanker Task, a variant of the Eriksen Flanker task, measures response inhibition/conflict monitoring by assessing the ability to modulate responses under congruent versus incongruent stimulus contexts. The Toolbox Dimensional Change Card Sort Task measures cognitive flexibility.[10,11] Each of the Toolbox tasks produces multiple scores, some of which are adjusted based on participant demographics, including raw scores, uncorrected standard scores, and age-corrected standard scores.[12] Age-corrected task scores were used in our analyses.

Rey auditory verbal learning test

The Rey Auditory Verbal Learning Test (RAVLT) measures auditory learning, memory, and recognition. This test requires children to listen to and recall a list of 15 unrelated words over five learning trials. After learning the initial list, a distractor list of 15 words is presented, and the child is asked to recall as many words from this second list as possible. Then, recall of the initially learned list is reassessed. Finally, recall following a 30-minute delay, during which participants engage in other non-verbal tasks, allows for the assessment of long-term retention.

Little man task

This task engages visual-spatial processing, specifically mental rotation, with varying degrees of difficulty. It involves presenting a rudimentary male figure holding a briefcase in one hand in the middle of the screen. The figure may appear in one of four positions: right side up or upside down, and either facing the respondent or with his back to the respondent. The briefcase may be in either the right or left hand. Children should indicate by button press which hand is holding the briefcase.

© 2025 Nivins S et al. *JAMA Netw Open*.

Covariates and Mediators descriptions
Parents/caregivers retrospectively completed the Developmental History Questionnaire at the child's age of 9-10 years, as developed by the Adolescent Component of the National Comorbidity Survey.[13-15] This questionnaire collected comprehensive information, including the biological mother's age at the time of the child's birth, birth weight (reported in pounds and ounces), gestational age (in weeks), and sex assigned at birth (male/female). It also assessed physical health problems during pregnancy (e.g., diabetes (yes/no)), birth complications (e.g., jaundice (yes/no)), and asked about the biological mother's substance use (i.e., tobacco (yes/no), alcohol (yes/no)) and breastfeeding months (at least partly for six months of age -yes/no).

In addition, parents/caregivers completed the Family History Questionnaire, which assesses the lifetime occurrences of various psychological issues, including depression and emotional/mental problems.
We used separate Multiple Correspondence Analysis (MCA) to reduce the dimensionality of maternal and neonatal characteristics. MCA is a form of exploratory factor analysis designed to handle categorical data, analogous to how PCA identifies latent variables in continuous data.[16] Each dimension generated by MCA offers a higher explanatory capacity than any single variable in the dataset, and can be represented as a continuous numerical scale, allowing for direct linear comparisons with biometric characteristics.

There is currently no universally accepted criterion for determining the number of dimensions to retain in MCA. However, studies have retrospectively used either an eigenvalue (inertia) threshold of > 0.1,[17-19] or considered only the first two dimensions, as they capture maximum variance.[19,20] We preferred the later approach and retained the first two dimensions (both with eigen values > 0.1), which accounted for 28% and 42% of the total variance for maternal and neonatal characteristics, respectively **(eFigure 1)**.

MCA was performed using the FactoMineR package in R.[21]

Socioeconomic status
SES was defined as the first principal component from a probabilistic PCA, capturing 65% of the variance in total household income, highest parental education, and neighbourhood quality. Children missing more than one of these SES measures were excluded. Household income was determined by the combined annual income of all family members over the past 12 months, categorized as less than $49,999 (1), $50,000–74,999 (2), $75,000–99,999 (3); $100,000–199,999 (4); and greater than $200,000 (5). Parental education was categorized into middle school or less (1), some high school (2), high school graduate (3), some college/associate degree (4), bachelor's degree (5), master's degree (6), or professional degree (7). The neighbourhood quality was determined using the area deprivation index, calculated from the American Community Survey using the address of the primary residency.[22] The SES composite and each subcomponent were normalized (mean=0, SD=1). The sample distribution of the SES is plotted in **eFigure 2**.

Genotyping, quality control, and imputation
Saliva samples were collected from all the children at 9-10 years and genotyped using Rutgers University Cell and DNA repository using the Smokescreen array consisting of 646,247 genetic variants.[23] Quality control, imputation, and genetic PCA were performed by the National Bioinformatics Infrastructure Sweden (NBIS). The following pre-processing steps were conducted. Briefly, single nucleotide polymorphisms (SNPs) with call rates < 98% or minor allele frequencies (MAFs) < 1% were excluded before imputation. Individuals with high rates of missingness (> 2%) and absolute autosomal heterozygosity >0.2 were excluded, resulting in 10,069 children and 430,622 genetic variants. Haplotypes were prephased using SHAPEIT2, and genetic markers were imputed using IMPUTE4 software.

We utilized the 1000 Genomes haplotypes—Phase 3 integrated variant set release in NCBI build 37 (hg19) coordinates as reference populations. This dataset consists of 2504 samples and 5008 haplotypes from Europeans, Africans, East Asians, Southern Asians, and Americans (https://mathgen.stats.ox.ac.uk/impute/1000GP_Phase3.html). We used this imputation since it provides better concordance in diverse human populations.[24,25] After that, genotypes with an INFO score < 0.3 or MAF < 0.001% were excluded, which yielded 40,637,119 SNPs in a total of 10,069 children.
The PCA module, as implemented in RICOPILI,[26] was used to check for outliers and control population structure. SNPs were pruned so that there was little linkage disequilibrium (LD) between SNPs ($R2 < 0.2$, 200 SNP window: Plink–indep-pairwise 200 100 0.2). LD pruning was repeated until 100 K SNPs were reached. The resulting SNPs were then projected into the PCA.[27,28]

© 2025 Nivins S et al. *JAMA Netw Open*.

cogPGS calculation
We created polygenic scores for cognitive performance (cogPGS) in each child using PRSice-2,[29] which involved summing the effect sizes of thousands of SNPs (weighted by the presence of effect alleles in each child). These SNPs were discovered by large genome-wide association studies (GWAS) on educational attainment, mathematical ability, and general cognitive ability.[30] Details regarding the effect sizes and p values of their SNPs can be assessed through the Social Science Genetics Association Consortium (https://www.thessgac.org/data).

We utilized the data provided by the consortium from a multitrait analysis of GWAS,[31] which, in our case, represents a joint polygenic score focused on a GWAS of cognitive performance and complemented by information from a GWAS on educational attainment, a GWAS on the highest-level math class completed, and a GWAS on self-reported math ability.[30] This joint analysis is ideal because pairwise genetic correlations of these traits were high,[30] and these GWAS had hundreds of thousands of individuals. Such a large sample size allows new studies to detect effects in samples of a few hundred individuals with 80% statistical power.

To construct the cogPGS, we performed clumping and pruning to remove nearby SNPs that are correlated with each other. The clumping sliding window was 250 kb, with the linkage disequilibrium clumping set to $r^2 > 0.25$. We included the weightings of all SNPs, regardless of their p-value from the GWAS ($p = 1.00$ threshold), resulting in 5255 SNPs. Finally, we normalized (mean=0, SD=1) the cogPGS to fairly compare their effects on different phenotypes. For the present study, we used cogPGS to represent the genetic predisposition to cognitive performance and included 20 genetic principal components (PCs) in the model to account for potential population stratification within the Add Health European-ancestry subsample. The use of the Add Health European-ancestry subsample was essential to minimize confounding due to population stratification, as genetic allele frequencies and their associations with phenotypes may vary across ancestries. By restricting the analysis to this subsample, we aimed to ensure that the cogPGS were appropriately calibrated and interpretable within a more genetically homogeneous group. Further, the inclusion of the first 20 genetic PCs further accounted for any residual population stratification within this subsample, enhancing the robustness of our findings. The sample distribution of the cogPGS is plotted in **eFigure 2**.

Pubertal development
Pubertal development was assessed using the Puberty Developmental Scale (PDS),[32] a self-reported or caregiver/parent-reported questionnaire designed to mimic the traditional Tanner staging assessment, but without the use of reference images. The PDS includes five items: three sex-neutral items (assessing skin changes, body hair growth, and height changes), two items specific to females (breast development and menarche), and two items specific to males (voice changes and facial hair growth). All items, except menarche, are rated on a 4-point scale (1=has not yet begun, 2=has barely begun, 4=definitely underway, 5=seems complete), with higher scores indicating more advanced pubertal development. We preferred parents/caregivers report of the PDS, as they show stronger correlations with clinician-assessed pubertal status,[33] and used total PDS scores in our analyses.

© 2025 Nivins S et al. *JAMA Netw Open*.

Race and Ethnicity
Parents/caregivers were asked to report the child's race and ethnicity using the Parent Demographic Survey. They could select from the following categories: 1. White; 2. Black/African American; 3. American Indian/Native American; 4. Alaska Native; 5. Native Hawaiian; 6. Guamanian; 7. Samoan; 8. Other Pacific Islander; 9. Asian Indian; 10. Chinese; 11. Filipino; 12. Japanese; 13. Korean; 14. Vietnamese; 15. Other Asian; 16. Other Race; 17. Refuse to Answer; and 18. Don't Know.

In the same Parent Demographic Survey parents/caregivers were also asked to report whether they considered the child to be Hispanic/Latino/Latina. Based on their responses, we categorized participants into the following groups, combining race and ethnicity classifications consistent with prior studies: Asian: Categories 9–15; Black: Category 2; Hispanic; Native American: Categories 3–4; Pacific Islander: Categories 5–8; White: Category 1; Other: Categories 16–18.

Statistical Analysis

Hierarchical regression analysis was employed to systematically examine the relationship between gestational age and cognitive outcomes while accounting for multiple variables. This approach allows for the sequential introduction of variable blocks, enabling the assessment of each block's unique contribution to the model. By first controlling for socio-demographic and genetic factors (Model 1), then adding maternal characteristics (Model 2), and finally incorporating additional child characteristics (Model 3), we can isolate the effects of preterm birth, and early-term birth on cognitive outcomes. This method provides a more nuanced understanding of the complex interplay between biological, genetic, and environmental factors influencing cognitive development in children born at different gestational ages.

Stepwise hierarchical regression analysis was used to investigate the relationship between gestational age and cognitive outcomes at 9-10 years of age. The model was first adjusted for child age, sex, SES, cogPGS, the first 20 PCs as fixed effects, with scanner sites as random effects (Model 1). Each subsequent model built upon the previous one: Model 2 added maternal characteristics, including maternal age, placental problems, hypertensive disorders, diabetes, infections during pregnancy, mode of delivery, alcohol or smoking during pregnancy, and mental health status (depression or anxiety) during pregnancy. Model 3 included Model 1 and Model 2 variables along with child characteristics, such as puberty status.

Criteria used for diagnosing ID
Children with Intellectual Developmental Disorder (IDD) were excluded using the NIH Toolbox WISC-V Matrix Reasoning Total Scale Score (TSS), which assesses fluid reasoning and problem-solving abilities. A TSS of ≤ 3 was used as cutoff, corresponding to an estimated IQ of ≤ 70, a common threshold for IDD classification.

Criteria used for diagnosing ADHD
Children with Attention-Deficit/Hyperactivity Disorder (ADHD) were identified using the Kiddie Schedule for Affective Disorders and Schizophrenia (KSADS), a semi-structured diagnostic interview designed to assess current and past episodes of psychiatric disorders based on DSM criteria. Specific modules focused on assessing core symptoms of inattention, hyperactivity, and impulsivity. Parent-reported information was used to evaluate the pervasiveness and impact of these symptoms across multiple settings, ensuring accurate identification of ADHD.

Distribution of outcome variables
The normality of continuous cognitive outcome variables was assessed by calculating the skewness and plotting histograms with overlaid normal curves. All cognitive outcome variables were found to be normally distributed, with skewness values ranging from -2 to +2. The skewness statistics for each cognitive outcome variable are provided in **eTable 1.**

eTable 1 Cognitive outcome measures of skewness

**Cognitive measures**	**Skewness**
Composite cognitive score	0.35
Vocabulary	0.56
Attention	0.43
Working memory	0.24
Executive function	1.05
Processing speed	-0.19
Episodic memory	0.64
Reading	1.13
Short delay recall	-0.57
Long delay recall	-0.45
Visuospatial accuracy	0.11
Visuospatial reaction time	-0.28

© 2025 Nivins S et al. *JAMA Netw Open*.

eTable 2 Detailed maternal and neonatal characteristics and childhood outcomes of those born preterm, early term and at term (N=5946)

Characteristic	Children born VPT (n=55)		Children born MPT (n=110)		Children born LP (n=454)		Children born ET (n=261)		Children born FT (n=5066)	
	Value	No.	Value	No.	Value	No.	Value	No.	Value	No.
**Maternal Characteristics**										
Age at delivery, mean (SD), years	28.0 (6.70)	55	30.1 (5.9)	110	30.5 (6.1)[c]	454	30.8 (5.9)[d]	261	29.5 (6.2)	5066
Mode of delivery, Caesarean, No. (%)	32 (58.2%)[a]	54	75 (68.2%)[b]	109	247 (54.4%)[c]	450	122 (46.7%)[d]	260	1560 (30.8%)	5005
Pregnancy complications, No. (%)										
Diabetes	4 (7.3%)	54	14 (12.7%)[b]	107	43 (9.5%)[c]	442	28 (10.7%)[d]	256	298 (5.8%)	4909
Hypertension disorders	17 (30.9%)[a]	54	30 (27.3%)[b]	108	85 (18.7%)[c]	441	43 (16.5%)[d]	255	351 (6.9%)	4893
Placenta previa or placental abruption	6 (10.9%)[a]	54	9 (8.2%)[b]	108	37 (8.1%)[c]	443	8 (3.0%)	25	98 (1.9%)	4915
Substance use during pregnancy, No. (%)										
Alcohol	0 (0%)	54	1 (0.9%)	108	9 (1.9%)	446	5 (1.9%)	257	154 (3.0%)	4936
Tobacco	3 (5.5%)	55	6 (5.5%)	107	25 (5.5%)	447	16 (6.1%)	259	257 (5.1%)	4951
Drugs	0 (0%)	53	0 (0%)	108	0 (0%)	442	2 (0.8%)	257	14 (0.3%)	4910
Mental health status during pregnancy, No. (%)										
Depressive problems	18 (32.7%)	52	25 (22.7%)	107	109 (24.0%)	429	72 (27.6%)	248	1132 (22.3%)	4817
Emotional/mental problems	14 (25.5%)	53	37 (33.6%)	107	158 (34.8%)	438	85 (32.6%)	256	1609 (31.8%)	4820
Maternal education level, No. (%)		55		110		454		261		5064
Middle school or less	0 (0%)		1 (0.9%)		1 (0.2%)		4 (1.5%)		70 (1.4%)	
Some high school	4 (7.2%)		3 (2.7%)		14 (3.1%)		12 (4.6%)		225 (4.4%)	
High school graduate	11 (20.0%)		18 (16.4%)		31 (6.8%)		14 (5.3%)		484 (9.6%)	

© 2025 Nivins S et al. *JAMA Netw Open*.

Some college/associate degree	15 (27.3%)		36 (32.7%)		136 (30.0%)		87 (33.3%)		1420 (28.0%)	
Bachelor's degree	14 (25.5%)		28 (25.5%)		146 (32.2%)		74 (28.4%)		1443 (28.5%)	
Master's degree	9 (16.4%)		20 (18.2%)		95 (20.9%)		53 (20.3%)		1060 (20.9%)	
Professional degree	2 (3.6%)		4 (3.6%)		31 (6.8%)		17 (6.5%)		362 (7.2%)	
**Neonatal Characteristics**										
Gestation length, mean (SD), weeks	29.0 (1.6)[a]	55	32.4 (0.5)[b]	110	35.3 (0.8)[c]	454	37.4 (0.5)[d]	261	40.0 (0.8)	5066
Sex assigned at birth, Boys	33 (60%)	55	64 (56.1%)	110	256 (53.0%)	454	155 (56.2%)	261	2727 (51.6%)	5066
Twin, No. (%)	23 (41.8%)[a]	55	60 (54.5%) [b]	110	229 (50.4%) [c]	454	65 (24.9%)[d]	261	333 (6.6%)	5046
Birthweight, mean (SD), kg	1.6 (0.6)[a]	55	1.8 (0.5)[b]	110	2.3 (0.5)[c]	454	2.8 (0.6)[d]	261	3.2 (0.5)	5066
Neonatal complications, No. (%)										
Jaundice	27 (49.1%)[a]	53	47 (42.7%)[b]	107	156 (34.4%)[c]	443	63 (24.1%)[d]	259	632 (12.5%)	4951
Respiratory support	38 (69.1%)[a]	55	59 (53.6%)[b]	105	127 (28.0%)[c]	435	30 (11.5%)[d]	250	206 (4.1%)	4951
Convulsions	0 (0%)	54	0 (0%)	104	0 (0%)	447	1 (0.3%)	258	7 (0.1%)	4980
Breast feeding of at least six months, yes	37 (67.3%)[a]	53	67 (60.9)[b]	106	268 (59.0%)[c]	444	139 (53.3%)[d]	257	2114 (41.7%)	4893
**Child Characteristics**										
Age, mean (SD), years	9.9 (0.7)	55	10.0 (0.6)	110	10.0 (0.6)	454	10.0 (0.6)[c]	261	9.9 (0.6)	5066
Puberty		52		104		427		252		4724
Boys										
Early (mean + 1SD)	5 (15.2%)		8 (13.1%)		54 (22.2%)[c]		24 (16.3%)		387 (14.9%)	
Time (mean)	20 (60.6%)		38 (62.3%)		142 (58.4%)		99 (67.3%)		1551 (59.7%)	
Late (mean – 1SD)	5 (15.2%)		10 (16.4%)		32 (13.2%)		21 (14.3%)		476 (18.3%)	
Girls										
Early (mean + 1SD)	7 (31.8%)		14 (28.6%)[b]		32 (15.2%)		17 (14.9%)		370 (15.0%)	

© 2025 Nivins S et al. *JAMA Netw Open*.

Time (mean)	13 (59.1%)		28 (57.1%)		136 (64.8%)		79 (69.3%)		1541 (62.5%)	
Late (mean – 1SD)	2 (0.9%)		6 (12.2%)		34 (16.2%)		12 (10.5%)		396 (16.1%)	
BMI (kg/m^2) [#]		55		110		454		261		5061
Boys										
Underweight	0 (0%)[a]		3 (4.9%)[b]		12 (4.9%)		2 (1.4%)		89 (3.4%)	
Normal	17 (51.5%)		36 (59.0%)		146 (60.1%)		85 (57.8%)		1510 (58.2%)	
Overweight	1 (3.0%)		6 (9.8%)		22 (9.0%)		23 (15.6%)		337 (13.0%)	
Obese	15 (45.5%)		16 (26.2%)		63 (25.9%)		37 (25.2%)		659 (25.4%)	
Girls										
Underweight	1 (4.5%)		4 (8.2%)		7 (3.3%)		4 (3.5%)		83 (3.4%)	
Normal	10 (45.5%)		24 (49.0%)		125 (59.5%)		68 (59.6%)		1514 (61.4%)	
Overweight	3 (13.6%)		8 (16.3%)		27 (12.9%)		16 (14.0%)		319 (12.9%)	
Obese	8 (36.4%)		13 (26.5%)		51 (24.3%)		26 (22.8%)		547 (22.2%)	
Race/Ethnicity		54		109		453		259		5047
Asian	1 (1.8%)		2 (1.8%)		23 (5.0%)		16 (6.1%)		296 (5.8%)	
Black	12 (21.8%)		19 (17.3%)		66 (14.5%)		31 (11.9%)		793 (15.7%)	
Hispanic	10 (18.2%)		28 (25.0%)		78 (17.2%)		51 (19.5%)		983 (19.4%)	
Native American	1 (1.8%)		0 (0%)		16 (3.5%)		5 (1.9%)		106 (2.1%)	
Pacific Islander	0 (0%)		1 (0.9%)		4 (0.8%)		1 (0.3%)		1 (0.01%)	
White	30 (54.5%)		59 (53.6%)		263 (57.9%)		153 (58.6%)		2779 (54.9%)	
Other[†]	0 (0%)		0 (0%)		3 (0.6%)		2 (0.8%)		76 (1.5%)	
Annual Household income, $		53		100		431		243		4694

© 2025 Nivins S et al. *JAMA Netw Open*.

≤ 49,999	23 (41.8%)[a]		30 (27.3%)		85 (18.7%)[c]		59 (22.6%)[d]		1275 (25.2%)	
50,000 – 74,999	11 (20.0%)		11 (10.0%)		65 (14.3%)		31 (11.9%)		633 (12.5%)	
75,000 – 99,999	4 (7.2%)		14 (12.7%)		70 (15.4%)		38 (14.6%)		707 (14.0%)	
1,00,000 – 1,99,999	14 (25.5%)		33 (30.0%)		156 (34.4%)		80 (30.7%)		1508 (29.8%)	
≥ 2,00,000	1 (1.8%)		12 (10.9%)		55 (12.1%)		35 (13.4%)		571 (11.3%)	

Data are expressed as mean (SD) or n (%) of study-sample unless otherwise indicated. 'No.' indicates the number of children included in the analysis for each variable.
Abbreviations: VPT, very preterm (28 to 31 completed weeks); MPT, moderate preterm (32 to 33 completed weeks); LP, late preterm (34 to 36 completed weeks); ET, early term (37 to 38 completed weeks); FT, full-term (≥ 39 completed weeks); and BMI, body mass index.

[#]BMI values were subsequently converted to age- and sex-specific percentiles based on the World Health Organization Child Growth Standards into underweight (less than 5th percentile), normal weight (between 5th and less than 85th percentile), overweight (between 85th and less than 95th percentile), and obese (95th percentile or above).
[†]Other is defined as category where parents/caregivers report the child's race/ethnicity as other race, refused to answer, or don't know.
[a]p < .05 for comparison between children born very preterm and at full-term; [b]p < .05 for comparison between children born moderate preterm and at full-term; [c]p < .05 for comparison between children born late preterm and at full-term; [d]p < .05 for comparison between children born early term and at full-term.

© 2025 Nivins S et al. *JAMA Netw Open*.

eTable 3 Association between moderate preterm birth and cognitive outcomes in children aged 9-10 years (Model 2 and Model 3)

**Cognitive measures**	**Model 2**		**Model 3**	
	**MPT vs FT**		**MPT vs FT**	
	**R^2**	**β (95%CI)**	**R^2**	**β (95%CI)**
Composite cognitive score	0.26	-0.36***<br>(-0.53 - -0.20)	0.26	-0.36***<br>(-0.53 - -0.19)
**NIH Toolbox**				
Vocabulary	0.25	-0.41***<br>(-0.58 - -0.24)	0.25	-0.41***<br>(-0.58 - -0.25)
Attention	0.05	-0.06<br>(-0.25 – 0.13)	0.05	-0.06<br>(-0.25 – 0.13)
Working memory	0.14	-0.25**<br>(-0.43 – -0.07)	0.14	-0.25**<br>(-0.43 – -0.07)
Executive function	0.06	-0.03<br>(-0.22 – 0.16)	0.07	-0.03<br>(-0.21 – 0.16)
Processing speed	0.06	-0.06<br>(-0.25 – 0.13)	0.06	-0.06<br>(-0.25 – 0.12)
Episodic memory	0.07	-0.30**<br>(-0.49 – -0.11)	0.07	-0.30**<br>(-0.49 – -0.11)
Reading	0.15	-0.20*<br>(-0.38 – -0.02)	0.15	-0.20*<br>(-0.38 – -0.02)
**Rey Auditory Verbal Learning Test**				
Short delay recall	0.10	-0.33***<br>(-0.51 – -0.14)	0.10	-0.31***<br>(-0.51 – -0.14)
Long delay recall	0.10	-0.26***<br>(-0.44 – -0.08)	0.10	-0.26**<br>(-0.44 – -0.07)
**Little Man Task**				
Visuospatial accuracy	0.13	-0.04<br>(-0.23 – 0.14)	0.13	-0.04<br>(-0.22 – 0.14)
Visuospatial reaction time	0.04	-0.19<br>(-0.38 – 0.01)	0.04	-0.19<br>(-0.38 – 0.001)

Abbreviations: MPT, moderately preterm; FT, full-term. *p < .05; **p < .01; and ***p < .001.

Model 1 was adjusted for child age, sex, SES, cogPGS, the first 20 PCs as fixed effects, with scanner sites as random effects.
Model 2: (Model 1 + maternal factors) – Used first two dimensions based on multiple correspondence analysis (MCA) analysis.
Model 3: (Model 2 + puberty scores)

© 2025 Nivins S et al. *JAMA Netw Open*.

eTable 4 Association between SES and cogPGS on cognitive outcomes in the overall cohort

Cognitive outcomes	R^2	cogPGS		SES	
		β (95%CI)	p	β (95%CI)	p
Composite cognitive score	0.25	0.14 (0.12 – 0.17)	< .001[a]	0.26 (0.24 - 0.29)	< .001[a]
**NIH Toolbox**					
Vocabulary	0.24	0.15 (0.12 – 0.18)	< .001[a]	0.23 (0.20 - 0.26)	< .001[a]
Attention	0.05	0.03 (0.001 – 0.06)	.04	0.11 (0.08 - 0.14)	< .001[a]
Working memory	0.13	0.09 (0.06 – 0.12)	< .001[a]	0.21 (0.18 - 0.24)	< .001[a]
Executive function	0.06	0.04 (0.01 – 0.07)	.01	0.13 (0.10 - 0.16)	< .001[a]
Processing speed	0.05	0.03 (0.001 – 0.06)	.03	0.04 (0.01 - 0.08)	.01
Episodic memory	0.07	0.08 (0.05 – 0.11)	< .001[a]	0.11 (0.08 - 0.14)	< .001[a]
Reading	0.15	0.14 (0.11 – 0.17)	< .001[a]	0.23 (0.20 - 0.26)	< .001[a]
**Rey Auditory Verbal Learning Test**					
Short delay recall	0.10	0.08 (0.05 – 0.11)	< .001[a]	0.13 (0.10 - 0.16)	< .001[a]
Long delay recall	0.10	0.08 (0.05 – 0.11)	< .001[a]	0.14 (0.11 - 0.17)	< .001[a]
**Little Man Task**					
Visuospatial accuracy	0.13	0.07 (0.04 – 0.10)	< .001[a]	0.16 (0.13 - 0.19)	< .001[a]
Visuospatial reaction time	0.04	0.04 (0.01 – 0.07)	.01	0.08 (0.05 - 0.12)	< .001[a]

Abbreviations: cogPGS, polygenic scores for cognitive performance; SES, socioeconomic status.

The model was adjusted for child age, sex, SES, cogPGS, the first 20 PCs as fixed effects, with scanner sites as random effects (Model 1).
[a]P-values that remain statistically significant after applying multiple comparison corrections.

© 2025 Nivins S et al. *JAMA Netw Open*.

eTable 5 Effect of sex on the association between moderate preterm birth and cognitive outcomes

**Cognitive outcomes**	**Model 1**<br>**GA group (MPT vs FT) x sex**
	**β (95%CI)**
Composite cognitive score	0.003 (-0.33 – 0.33)
**NIH Toolbox**	
Vocabulary	0.10 (-0.23 – 0.43)
Attention	-0.16 (-0.54 – 0.21)
Working memory	-0.04 (-0.40 – 0.31)
Executive function	-0.001 (-0.37 – 0.37)
Processing speed	-0.09 (-0.46 – 0.29)
Episodic memory	0.08 (-0.29 – 0.45)
Reading	0.02 (-0.33 – 0.38)
**Rey Auditory Verbal Learning Test**	
Short delay recall	0.002 (-0.36 – 0.37)
Long delay recall	0.10 (-0.27 – 0.46)
**Little Man Task**	
Visuospatial accuracy	-0.09 (-0.45 – 0.27)
Visuospatial reaction time	0.14 (-0.24 – 0.52)

Abbreviations: GA, gestational age groups; MPT, moderately preterm; FT, full-term.

Model 1: adjusted for age, sex, socio-economic status, polygenic scores for cognitive ability, 20 PCs (first 20 principal components for ancestry components), along with an interaction terms of sex, and with scanner sites as random effects.

© 2025 Nivins S et al. *JAMA Netw Open*.

eTable 6 Association between moderate preterm and cognitive development at 9-10 years, excluding children who were not of European ancestry

**Cognitive outcomes**	**R^2**	**cogPGS**	**MPT (N=59) vs FT (n=2799)**
		**β (95%CI)**	**β (95%CI)**
Composite cognitive score	0.15	0.18***<br>(0.14 – 0.22)	-0.32*<br>(-0.56 - -0.07)
**NIH Toolbox**			
Vocabulary	0.10	0.17***<br>(0.13 – 0.21)	-0.37**<br>(-0.62 - -0.12)
Attention	0.02	0.01<br>(-0.03 – 0.05)	-0.07<br>(-0.33 – 0.20)
Working memory	0.07	0.12***<br>(0.08 – 0.16)	-0.27*<br>(-0.52 – -0.01)
Executive function	0.03	0.03<br>(-0.01 – 0.07)	0.11<br>(-0.15 – 0.37)
Processing speed	0.05	0.04<br>(-0.004 – 0.08)	-0.04<br>(-0.29 – 0.22)
Episodic memory	0.04	0.08***<br>(0.04 – 0.13)	-0.22<br>(-0.47 - 0.04)
Reading	0.08	0.15***<br>(0.11 – 0.20)	-0.16<br>(-0.42 - 0.09)
**Rey Auditory Verbal Learning Test**			
Short delay recall	0.06	0.12***<br>(0.08 – 0.16)	-0.26*<br>(-0.51 - -0.001)
Long delay recall	0.07	0.10***<br>(0.06 – 0.14)	-0.27*<br>(-0.52 - -0.01)
**Little Man Task**			
Visuospatial accuracy	0.11	0.09<br>(0.05 – 0.13)	-0.05<br>(-0.30 - 0.20)
Visuospatial reaction time	0.03	0.05<br>(0.003 – 0.09)	-0.37<br>(-0.63 - 0.10)

Abbreviations: cogPGS, polygenic scores for cognitive performance; MPT, moderately preterm; FT, full-term.
*p < .05; **p < .01; ***p < .001.

© 2025 Nivins S et al. *JAMA Netw Open*.

eTable 7 Association between moderate preterm and cognitive development at 9-10 years, excluding children with intellectual disability or ADHD

**Cognitive measures**	**MPT (n=99) vs FT (n=4494)**	
	**R^2**	**β (95%CI)**
Composite cognitive score	0.25	-0.39***<br>(-0.57 - -0.21)
**NIH Toolbox**		
Vocabulary	0.26	-0.40***<br>(-0.58 - -0.23)
Attention	0.04	-0.04<br>(-0.24 - 0.16)
Working memory	0.12	-0.25**<br>(-0.44 - -0.06)
Executive function	0.06	-0.03<br>(-0.23 - 0.17)
Processing speed	0.05	-0.03<br>(-0.23 - 0.17)
Episodic memory	0.07	-0.36***<br>(-0.56 - -0.17)
Reading	0.14	-0.18<br>(-0.37 - 0.01)
**Rey Auditory Verbal Learning Test**		
Short delay recall	0.09	-0.36***<br>(-0.55 - -0.16)
Long delay recall	0.08	-0.31**<br>(-0.50 - -0.11)
**Little Man Task**		
Visuospatial accuracy	0.11	-0.08<br>(-0.27 - 0.11)
Visuospatial reaction time	0.02	-0.23*<br>(-0.43 - -0.03)

Abbreviations: MPT, moderately preterm; FT, full-term. *p < .05; **p < .01; and ***p < .001.

The model was adjusted for child age, sex, SES, cogPGS, the first 20 PCs as fixed effects, with scanner sites as random effects.

© 2025 Nivins S et al. *JAMA Netw Open*.

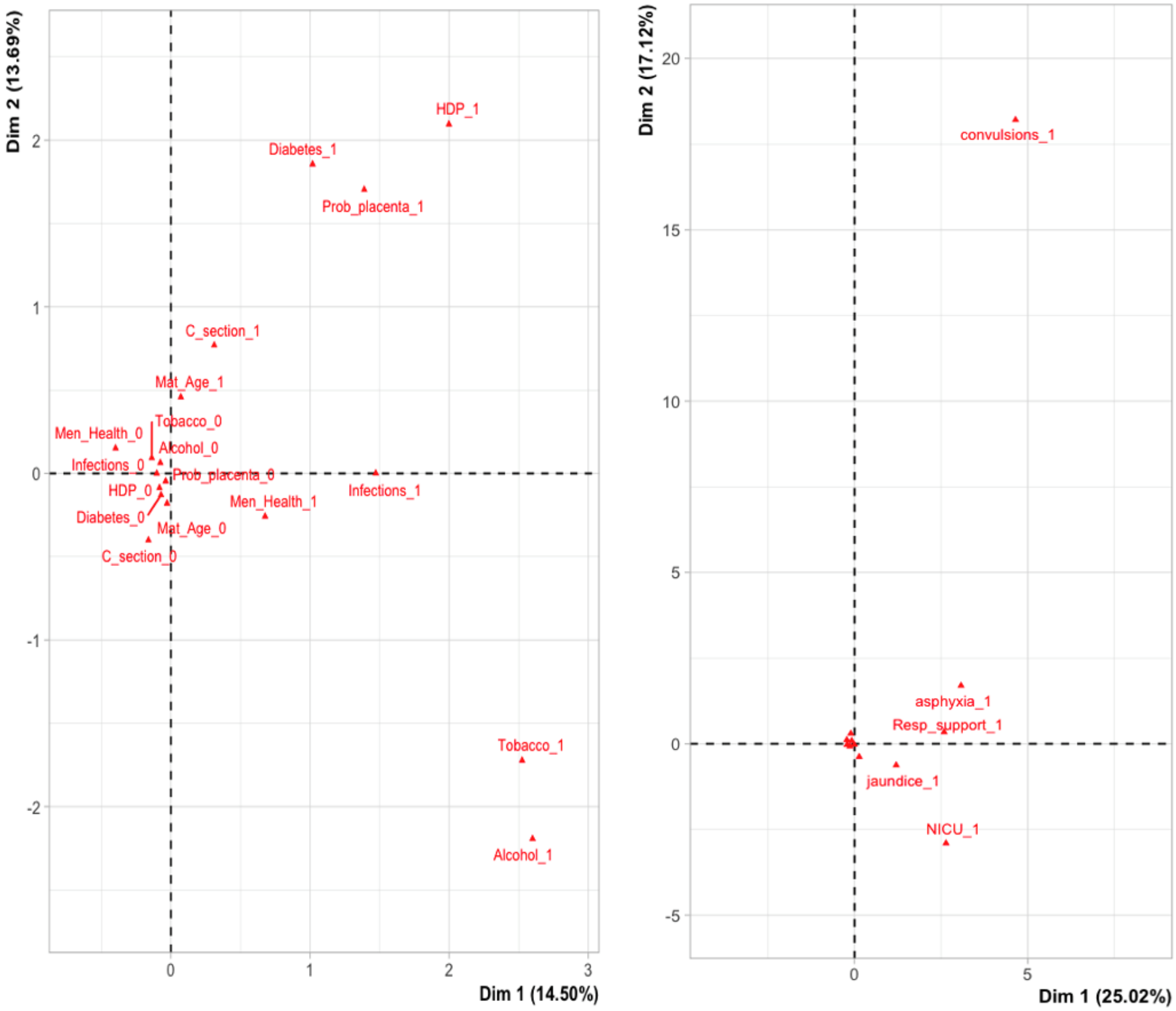

eFigure 1 Multiple correspondence analysis (MCA) factor map. Left, MCA of maternal characteristics showing two dimensions explaining 30% of the variance. Right, MCA of neonatal characteristics showing two dimensions explaining 50% of the variance. C_section, Casearian section; Men_health, mental health status; Mat_Age, maternal age; HDP, hypertensive disorders of pregnancy; Resp_support, respiratory support; NICU, admitted in neonatal care unit.

© 2025 Nivins S et al. *JAMA Netw Open*.

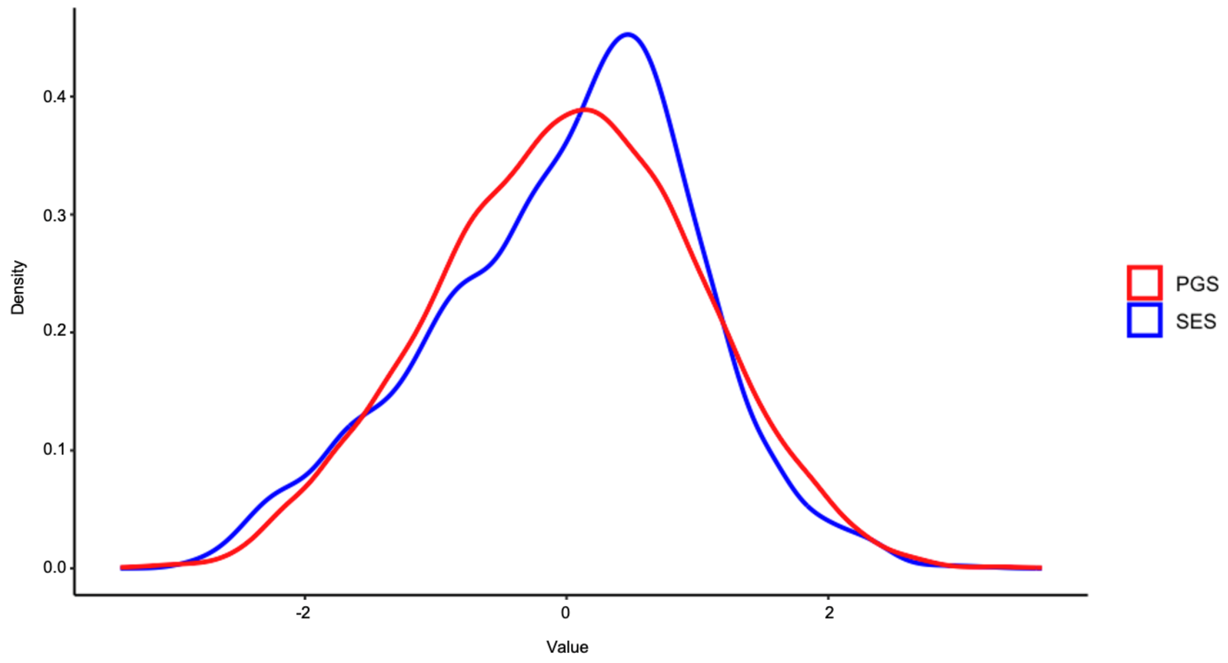

eFigure 2 Distributions of polygenic scores for cognitive performance (PGS) socioeconomic status (SES) in the included children (n=5946).

© 2025 Nivins S et al. *JAMA Netw Open*.

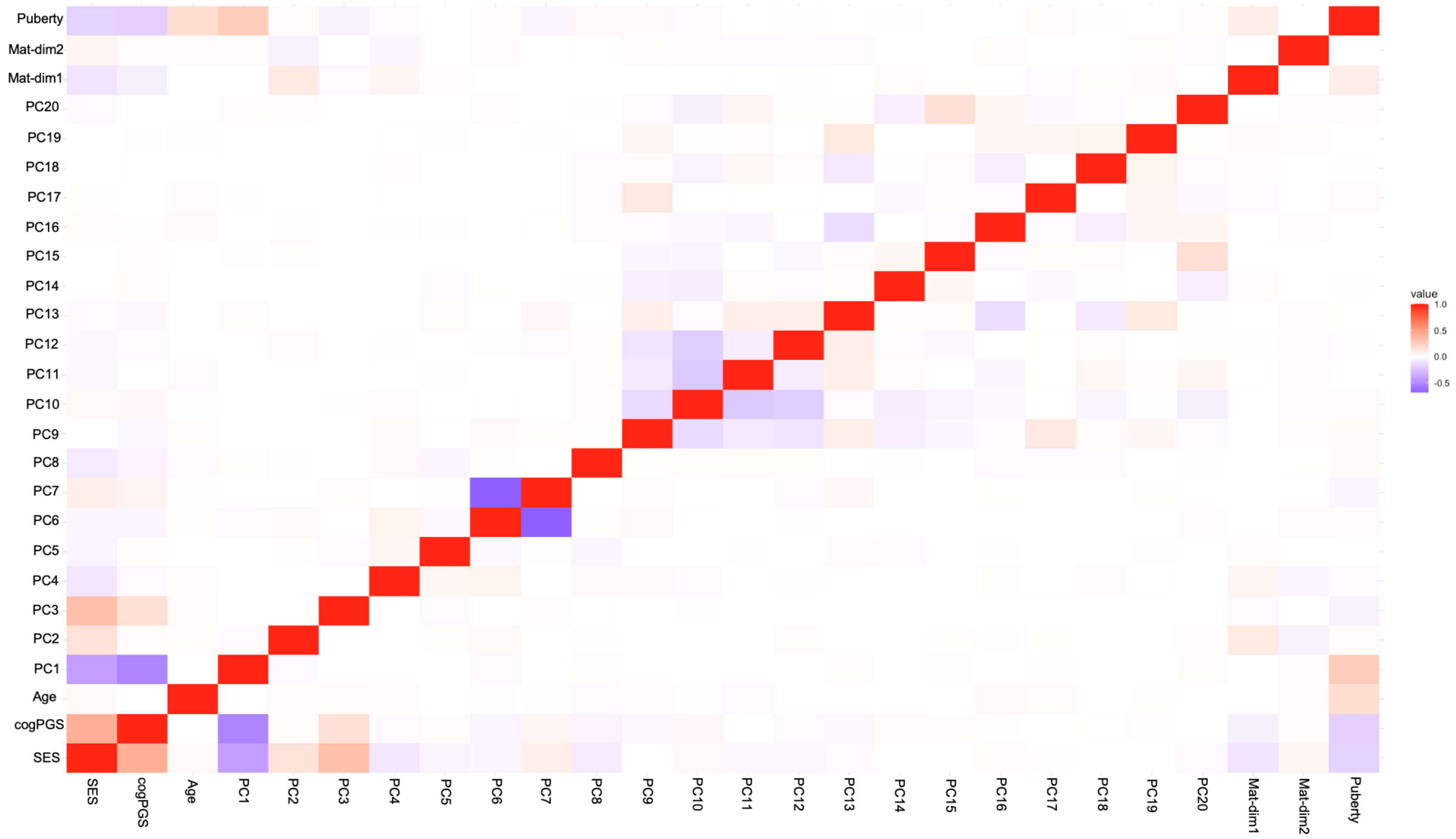

eFigure 3 Heat map of the correlation matrix between pairs of covariates. Darker colours indicate higher correlations. None of the correlations exceed 0.7.
Abbreviations: Mat-dim1 : Mat-dim2; first two dimensions of maternal characteristics; PC1:PC20, first 20 principal components of genetic ancestry; cogPGS, polygenic scores for cognitive performance (cogPGS); and SES, socioeconomic status.

© 2025 Nivins S et al. *JAMA Netw Open*.

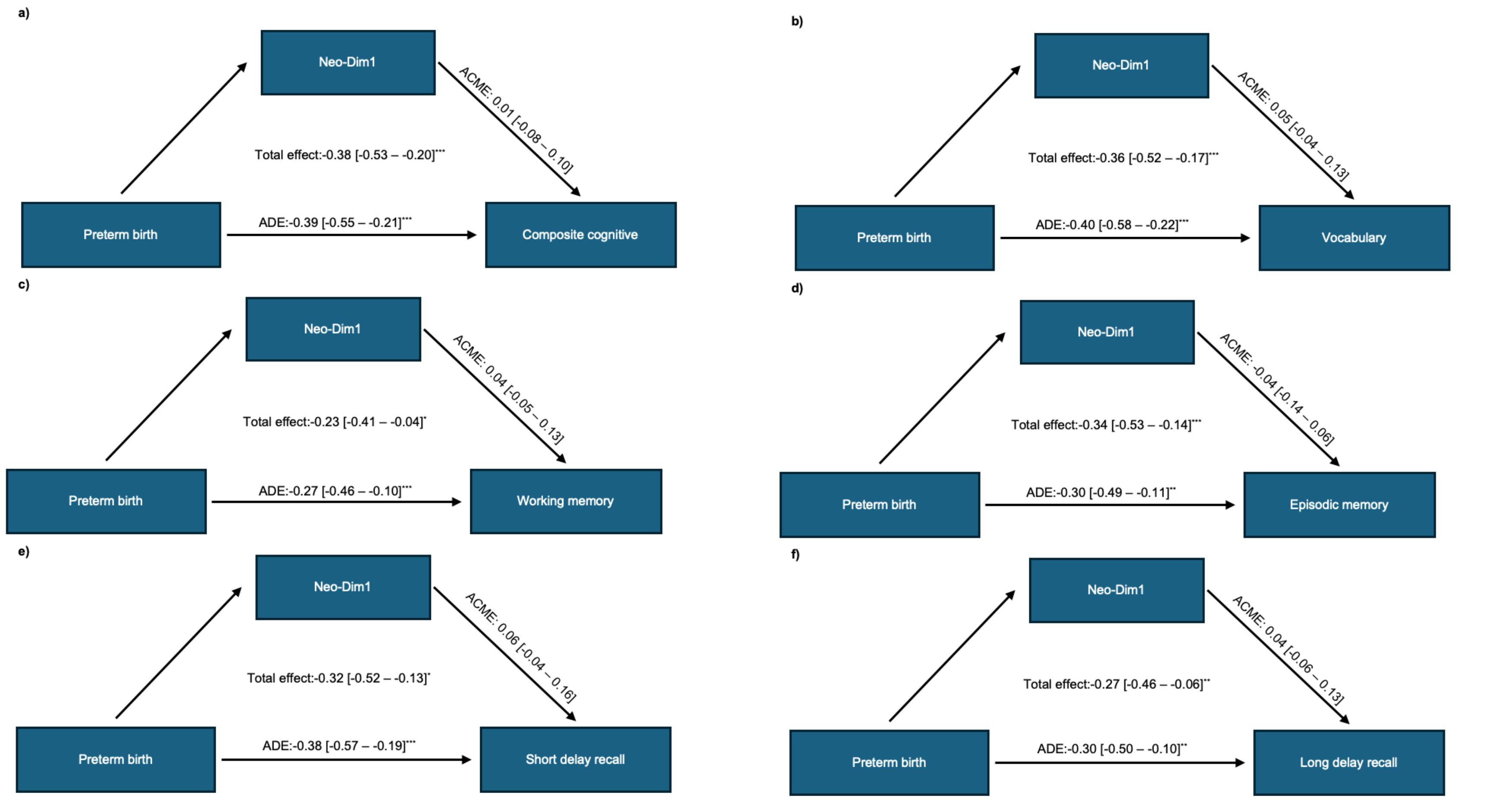

eFigure 4 Mediation analysis of first dimension of neonatal factors in cognitive outcomes after moderately preterm birth showing no mediation. Neo-Dim1 is the first dimension which captures ~25% of variance of neonatal characteristics.
Abbreviations: ACME, Average Causal Mediation Effects; ADE, Average Direct Effects.

© 2025 Nivins S et al. *JAMA Netw Open*.

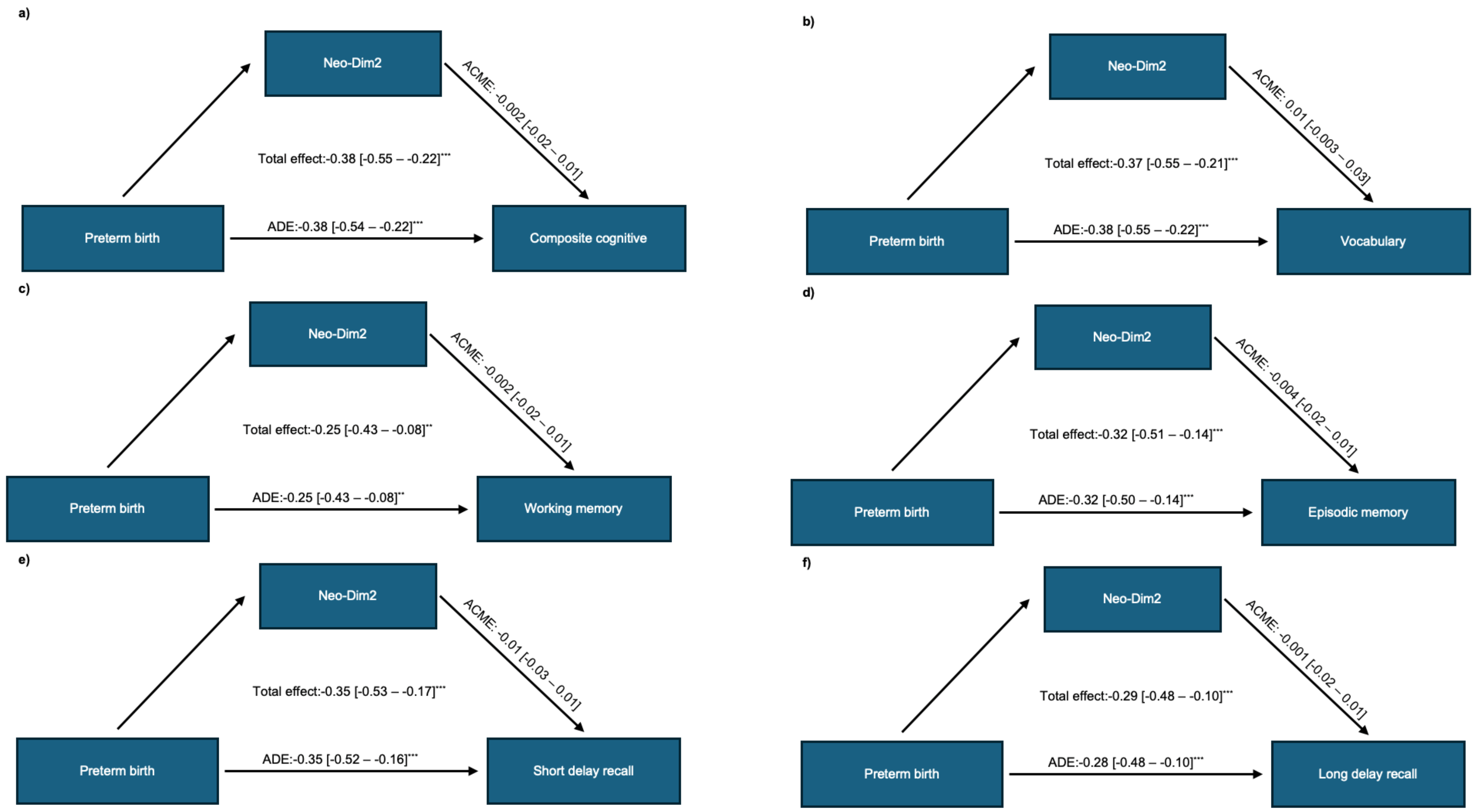

eFigure 5 Mediation analysis of second dimension of neonatal factors in cognitive outcomes after moderately preterm birth showing no mediation. Neo-Dim1 is the first dimension which captures ~17% of variance of neonatal characteristics.
Abbreviations: ACME, Average Causal Mediation Effects; ADE, Average Direct Effects.

© 2025 Nivins S et al. *JAMA Netw Open*.

eReferences

1. Bleck TP, Nowinski CJ, Gershon R, Koroshetz WJ. What is the NIH Toolbox, and what will it mean to neurology? : AAN Enterprises; 2013. p. 874-875.
2. Gershon RC, Wagster MV, Hendrie HC, Fox NA, Cook KF, Nowinski CJ. NIH toolbox for assessment of neurological and behavioral function. *Neurology*. 2013;80(11_supplement_3):S2-S6.
3. Hodes RJ, Insel TR, Landis SC, Research NBfN. The NIH toolbox: setting a standard for biomedical research. *Neurology*. 2013;80(11_supplement_3):S1-S1.
4. Gershon RC, Cook KF, Mungas D, et al. Language measures of the NIH toolbox cognition battery. *Journal of the International Neuropsychological Society*. 2014;20(6):642-651.
5. Gershon RC, Slotkin J, Manly JJ, et al. IV. NIH Toolbox Cognition Battery (CB): measuring language (vocabulary comprehension and reading decoding). *Monographs of the Society for Research in Child Development*. 2013;78(4):49-69.
6. Carlozzi NE, Beaumont JL, Tulsky DS, Gershon RC. The NIH toolbox pattern comparison processing speed test: normative data. *Archives of Clinical Neuropsychology*. 2015;30(5):359-368.
7. Carlozzi NE, Tulsky DS, Chiaravalloti ND, et al. NIH toolbox cognitive battery (NIHTB-CB): The NIHTB pattern comparison processing speed test. *Journal of the International Neuropsychological Society*. 2014;20(6):630-641.
8. Bauer PJ, Dikmen SS, Heaton RK, Mungas D, Slotkin J, Beaumont JL. III. NIH Toolbox Cognition Battery (CB): measuring episodic memory. *Monographs of the Society for Research in Child Development*. 2013;78(4):34-48.
9. Dikmen SS, Bauer PJ, Weintraub S, et al. Measuring episodic memory across the lifespan: NIH toolbox picture sequence memory test. *Journal of the International Neuropsychological Society*. 2014;20(6):611-619.
10. Zelazo PD, Anderson JE, Richler J, Wallner-Allen K, Beaumont JL, Weintraub S. II. NIH Toolbox Cognition Battery (CB): Measuring executive function and attention. *Monographs of the Society for Research in Child Development*. 2013;78(4):16-33.
11. Zelazo PD, Anderson JE, Richler J, et al. NIH Toolbox Cognition Battery (CB): Validation of executive function measures in adults. *Journal of the International Neuropsychological Society*. 2014;20(6):620-629.
12. Casaletto KB, Umlauf A, Beaumont J, et al. Demographically corrected normative standards for the English version of the NIH Toolbox Cognition Battery. *Journal of the International Neuropsychological Society*. 2015;21(5):378-391.
13. Kessler RC, Avenevoli S, Costello EJ, et al. Design and field procedures in the US National Comorbidity Survey Replication Adolescent Supplement (NCS-A). *International Journal of Methods in Psychiatric Research*. 2009/06/01 2009;18(2):69-83. doi:https://doi.org/10.1002/mpr.279
14. Kessler RC, Avenevoli S, Costello EJ, et al. National Comorbidity Survey Replication Adolescent Supplement (NCS-A): II. Overview and Design. *Journal of the American Academy of Child & Adolescent Psychiatry*. 2009/04/01/ 2009;48(4):380-385. doi:https://doi.org/10.1097/CHI.0b013e3181999705
15. Merikangas KR, He JP, Burstein M, et al. Lifetime prevalence of mental disorders in U.S. adolescents: results from the National Comorbidity Survey Replication--Adolescent Supplement (NCS-A). *J Am Acad Child Adolesc Psychiatry*. Oct 2010;49(10):980-9. doi:10.1016/j.jaac.2010.05.017
16. Greenacre M, Blasius J. *Multiple correspondence analysis and related methods*. Chapman and Hall/CRC; 2006.
17. Florensa D, Mateo-Fornés J, Solsona F, et al. Use of Multiple Correspondence Analysis and K-means to Explore Associations Between Risk Factors and Likelihood of Colorectal Cancer: Cross-sectional Study. *J Med Internet Res*. Jul 19 2022;24(7):e29056. doi:10.2196/29056
18. Kim DH, Rajaguru V, Kim B, et al. Association of behavior pattern with overweight and obesity in South Korean adults–A multi correspondence analysis (KNHANES-2018–2020). *PLOS Global Public Health*. 2023;3(9):e0002384. doi:10.1371/journal.pgph.0002384
19. Costa PS, Santos NC, Cunha P, Cotter J, Sousa N. The Use of Multiple Correspondence Analysis to Explore Associations between Categories of Qualitative Variables in Healthy Ageing. *J Aging Res*. 2013;2013:302163. doi:10.1155/2013/302163
20. Gifi A. *Nonlinear multivariate analysis*. vol 8. Wiley Chichester; 1990.
21. Lê S, Josse J, Husson F. FactoMineR: An R Package for Multivariate Analysis. *Journal of Statistical Software*. 03/18 2008;25(1):1 - 18. doi:10.18637/jss.v025.i01
22. Kind AJH, Jencks S, Brock J, et al. Neighborhood socioeconomic disadvantage and 30-day rehospitalization: a retrospective cohort study. *Annals of internal medicine*. 2014;161(11):765-774. doi:10.7326/M13-2946
23. Baurley JW, Edlund CK, Pardamean CI, Conti DV, Bergen AW. Smokescreen: a targeted genotyping array for addiction research. *BMC genomics*. 2016;17(128):145-145. doi:10.1186/s12864-016-2495-7
24. Corresponding GPC. An integrated map of genetic variation from 1,092 human genomes. *Nature*. 2012;491(7422):56-65.
25. Howie B, Donnelly P, Marchini J. 1,000 Genomes haplotypes—Phase 3 integrated variant set release in NCBI build 37 (hg19) coordinates. 2015.
26. Lam M, Awasthi S, Watson HJ, et al. RICOPILI: Rapid Imputation for COnsortias PIpeLIne. *Bioinformatics*. Feb 1 2020;36(3):930-933. doi:10.1093/bioinformatics/btz633
27. Nielsen TT, Duan J, Levey DF, et al. Disentangling the shared genetics of ADHD, cannabis use disorder and cannabis use and prediction of cannabis use disorder in ADHD. *medRxiv*. 2024:2024.02. 22.24303124.
28. Zhou H, Kember RL, Deak JD, et al. Multi-ancestry study of the genetics of problematic alcohol use in over 1 million individuals. *Nature Medicine*. 2023/12/01 2023;29(12):3184-3192. doi:10.1038/s41591-023-02653-5
29. Choi SW, O'Reilly PF. PRSice-2: Polygenic Risk Score software for biobank-scale data. *Gigascience*. 2019;8(7)doi:10.1093/gigascience/giz082
30. Lee JJ, Wedow R, Okbay A, et al. Gene discovery and polygenic prediction from a genome-wide association study of educational attainment in 1.1 million individuals. *Nature genetics*. 2018;50(8):1112-1121.
31. Turley P, Walters RK, Maghzian O, et al. Multi-trait analysis of genome-wide association summary statistics using MTAG. *Nature genetics*. 2018;50(2):229-237.
32. Petersen AC, Crockett L, Richards M, Boxer A. A self-report measure of pubertal status: Reliability, validity, and initial norms. *Journal of youth and adolescence*. 1988;17(2):117-133.
33. Rasmussen AR, Wohlfahrt-Veje C, Tefre de Renzy-Martin K, et al. Validity of self-assessment of pubertal maturation. *Pediatrics*. 2015;135(1):86-93.

© 2025 Nivins S et al. *JAMA Netw Open*.
