# Supplementary material for: Gestational Age and Cognitive Development in Childhood
Source: JAMA Netw Open. 2025 Apr 14;8(4):e254580. doi: 10.1001/jamanetworkopen.2025.4580 (PMC11997729; doi:10.1001/jamanetworkopen.2025.4580)
Supplement: Supplement 2. — Statistical Analysis Plan [file jamanetwopen-e254580-s002.pdf]

Statistical Analysis Plan

Title: Preterm birth on cognitive outcomes

Prepared by Samson Nivins with feedback and inputs from Ulrika Arden

Final draft prepared on
2024 – 06 - 18

Table of Contents

INTRODUCTION ---------- 3

STUDY OBJECTIVES ---------- 5

HYPOTHESIS ---------- 5

METHODS ---------- 6

PARTICIPANTS ---------- 6
NEUROCOGNITIVE MEASURES ---------- 6
REY AUDITORY VERBAL LEARNING TEST ---------- 7
LITTLE MAN TASK ---------- 7

OUTCOMES ---------- 9

PRIMARY OUTCOME ---------- 9
SECONDARY OUTCOMES ---------- 9
EXPOSURES ---------- 9

STATISTICS ---------- 10

PLANNED FIGURES AND TABLES ---------- 11

REFERENCE ---------- 12

APPENDIX ---------- 14

SOCIOECONOMIC STATUS ---------- 14
POLYGENIC SCORE DERIVATION AND ANALYSES ---------- 14
GENOTYPING, QUALITY CONTROL, AND IMPUTATION ---------- 14
COGPGS CALCULATION ---------- 15

Introduction

Preterm birth, defined as birth before 37 weeks of gestation, is a leading cause of child morbidity and mortality. According to the World Health Organization, ~13 million babies are born preterm each year globally, which equates to more one premature birth for every ten deliveries.[1] Specifically, in Nordic countries, the prevalence of preterm birth is estimated to be around 5.8%.[2] Due to rapid advancements in obstetrics and neonatal care, most of these preterm babies have a better survival rate;[3] however, they are more prone to cognitive deficits and neurodevelopmental disorders, such as ADHD,[4] which exerts a heavy burden on affected families, society and to the health care system.

Preterm birth is associated with poor cognitive abilities. However, studies so far have mainly focused on children born very preterm (< 32 weeks of gestation) or extremely preterm (< 28 weeks of gestation), even though children born moderate to late-preterm (32 – 36 weeks of gestation) make up a substantial proportion of preterm birth groups.[5,6] Compared to those born at term, children born moderate to late preterm are more prone to acute morbidity conditions such as respiratory distress, temperature instability, and neonatal hypoglycaemia.[7,8] Despite this, these babies have been overlooked by the perinatal community, with extensive research mainly focused on children born extremely/very preterm. Recent evidence suggests that those born moderate-to late preterm and early-term, also at high risk of cognitive difficulties compared to their term born peers, yet studies have produced inconsistent results. For example, some studies have shown lower cognitive functioning in the moderate-to late preterm group during childhood,[9-17] while others have shown no differences.[18,19]

In general, cognitive abilities is largely heritable, with heritability estimates reaching upto 70%. Interestingly, the heritability of general cognitive abilities increase significantly from childhood to early adulthood.[20] Prior studies focusing on contribution of the heritability and biological risk factors, such as preterm birth, have mostly adopted familial designs. Previous studies using sibling analysis have investigated the association between gestational age and intelligence quotient (IQ),[21,22] finding that these associations are independent of shared familial confounders and statistical covariates. While these studies provide initial insight into the effect of biological risk factors, such as preterm birth, accounting for genetic mechanisms underlying with IQ, they have several limitations. First, these designs require assumptions that can sometimes be violated, for example, siblings do not necessarily share 50% of their genes, nor do they necessarily inherit same risk alleles. Second, this approach cannot account for specific environmental effects, such as maternal factors. Therefore, distinguishing environmental factors and conducting separate genotyping would identify specific environmental contributions, which could be targets for improving cognitive outcomes.

Previous familial design studies focusing on association between gestational age and IQ, have primarily focused on general cognition and have not explored individual domains. It is known that children born preterm show delay in cognitive abilities, but majority of these children perform within normal range for general cognitive functioning as a group (i.e., they perform 0.5 - 1.0 SD below that of term-born peers).[23-26] But, specific functions such as attention, working memory, and processing speed are often delayed in this cohort,[25,27] have not been explored.

In addition, preterm birth is a syndrome where various causes might act synergistically to its manifestation.[28] Previous systematic and meta-analyses have assessed numerous non-genetic risk factors for preterm birth, such as obstetric and medical history, environmental exposures, and postnatal factors. Exploring the independent contribution of these risk factors on cognitive abilities could pave the way for risk-specific interventions and enhance our understanding of the mechanisms related to these outcomes.

Study objectives

The aims of this study are two-fold:

- Investigate the association between preterm and early-term births on cognitive outcomes during mid-childhood; and assess the extent to which these associations can be explained by unmeasured genetic and environmental factors.

- Investigate the association between weeks of gestation and cognitive outcomes during mid-childhood; and assess the extent to which these associations can be explained by unmeasured genetic and environmental factors.

Hypothesis

Children born preterm is independently associated with poor cognitive abilities, with larger effect size for lower gestational ages.

Methods

Participants

The neuroimaging and behavioural data used in this study will be obtained from the ABCD Study (data release 5.0; https://abcdstudy.org/; http://doi.org/10.15154/1523041), a longitudinal cohort of 11,875 children born between 2005 and 2009. These children were enrolled at ages 9-11 years from 21 research sites across the U.S. between 2016 and 2018,[29] with the intention of following them for a period of at least 10 years. This recruitment cohort closely matches the sociodemographic composition of the US population of 9-11-year-old children. Most of the children were enrolled through local elementary and charter schools at each data-collection site. A smaller portion was recruited through community outreach and word-of-mouth referrals outside of the school setting. Twins were identified and recruited from birth registries.[30,31]

During each visit, children accompanied by a parent/guardian, completed a series of measures. These included neurocognitive tests, mental and physical health questionnaires, environmental exposure data collection, providing biological specimens, and participating in brain imaging.[29,32-36] All were asked for an in-person assessment session for self- or parent-report of mentioned behavioural measures and for biological specimen collections once a year, with brain imaging conducted biannually. For this study, we will use data collected between 2016 and 2018.

Children were excluded if they were born extremely preterm (< 28 weeks of gestation) or had birth weight (< 1200 g), were not proficient in English, had any neurological problems, had a history of seizures, or had a contraindication to undergo brain MRI scans. All children and their parents/guardians provided informed written consent/assent for participation, and the central Institutional Review Board at the University of California, San Diego approved the study protocols. All the research methods were performed in accordance with the relevant guidelines and regulations.

Neurocognitive measures

The neurocognitive battery was designed to be completed in 70 min. Participants first completed the Snellen vision chart as a measure of visual acuity. Legal blindness (with vision correction) was a study exclusion.

A brief handedness inventory, consisting of four self-report questions, was also administered.

The neurocognitive testing battery, comprised of ten measures, was then initiated. All tests were administered using an iPad with one-on-one monitoring by a research assistant. The neurocognitive

battery consists of seven different tasks that cover episodic memory, executive function, attention, working memory, processing speed, and language abilities, and was normed for samples between the ages of 3 and 85 years. The total administration time for the NIH Toolbox Cognitive battery is approximately 35 min.

The Toolbox Picture Vocabulary Task measures language skills and verbal intellect. The Toolbox Oral Reading Recognition Task is a reading test that asks individuals to pronounce single words. The Toolbox Pattern Comparison Processing Speed Test is a measure of rapid visual processing. The Toolbox List Sorting Working Memory Test requires participants to use working memory to sequence task stimuli based on category membership and perceptual characteristics. The Toolbox Picture Sequence Memory Test was modelled after memory tests asking children to imitate a sequence of actions using props. The Toolbox Flanker Task, a variant of the Eriksen Flanker task, is a response inhibition/conflict monitoring task that measures the ability to modulate responding under congruent versus incongruent stimulus contexts. The Toolbox Dimensional Change Card Sort Task measures cognitive flexibility. Each of the Toolbox tasks produces several scores, some of which are adjusted based on participant demographics. All tasks provide raw scores, uncorrected standard scores, and age-corrected standard scores.
Age corrected task scores will be used in our analyses.

Rey auditory verbal learning test

The Rey Auditory Verbal Learning Test (RAVLT) measures auditory learning, memory, and recognition. A customized automated version, created through the Q-interactive platform of Pearson assessments was used. This test requires participates to listen to and recall a list of 15 unrelated words over five learning trials. Following initial learning of the list, a distractor list of 15 words is presented, and the participant is asked to recall as many words from this second list as he/she is able. Next, recall of the initially learned list is assessed. Recall following a 30-min delay (during which participants engage in other non-verbal tasks), permits longer term retention to be assessed.

Little man task

This task engages visual-spatial processing, specifically mental rotation, with varying degrees of difficulty. The task involves the presentation of a rudimentary male figure holding a briefcase in one hand in the middle of the screen. The figure may appear in one of four positions: right side up vs. upside down and either facing the respondent or with his back to the respondent. The briefcase may be in either the right or left hand. Respondents indicate by button press which hand is holding the briefcase.

Outcomes

Primary outcome

- Cognitive composite scores

Secondary outcomes

- Domains of cognitive scores

Exposures

Full-term 39 weeks of gestation and above

Early-term 37-38 weeks of gestation

Late-preterm 34 – 36 weeks of gestation

Moderate-preterm 32 – 34 weeks of gestation

Very preterm 28 – 32 weeks

Covariates

Age of a child

Sex assigned at birth

Socio-economic status (based on PCA – total household income, highest parental education, and neighbourhood quality) (See appendix – page 15)

Polygenic scores (See appendix – page 15)

20 Principal components to account for the possibility of population stratification within the Add Health European-ancestry subsample in the same model.

Maternal factors (Hypertension, diabetes, placental problems, maternal age, infections, C-section, alcohol consumptions, tobacco usage, and mental health problems).

Multiple correspondence analysis (MCA) will be applied and components with over 60% of variance will be used.

Neonatal factors (Dyspnea, convulsion, jaundice, oxygen support, NICU). MCA will be applied.

Child factors (puberty).

Scanner sites as recommended by ABCD

Statistics

Maternal and child characteristics will be summarized using means and standard deviations for continuous variables and frequencies with counts for categorical distributions. To compare the demographic and clinical characteristics between children born preterm, early-term and born at full-term, independent samples t-test and χ^2/Fisher exact tests will be employed. Missing covariates will be imputed through multiple imputations by chained equations with the predictive mean matching method using the MICE package in R.

Hierarchical logistic/linear regression model will be used to investigate the relationships between preterm birth and behavioural/brain outcomes. Model 1: Adjusted for age of child, sex, SES, Polygenic scores, 20 PCs, and scanner sites; Model 2: Model 1 + Maternal factors; and Model 3: Model 2 + Neonatal factors; Model4: Model 3 + child factors

Primary analyses:

- Linear regression model will be used to investigate whether the one-factor (intelligence) differ between preterm birth, early-term birth and full-term birth (presented as standardized beta and 95% CI)

Secondary analyses:

- Linear regression model will be used to investigate whether the general cognitive abilities, executive functions, and working memory performance differ between preterm birth and full-term birth (presented as standardized beta and 95% CI)

Exploratory analyses:

Sex and birthweight

Primary analysis will be uncorrected with statistical significance at 0.05. Secondary analysis will be adjusted for multiple comparison corrections.

All analysis will be carried out using R studio.

Planned Figures and Tables

Table 1 Demographics of maternal and child characteristics
Table 2 Results from the primary analysis
Table 3 Results from the weeks of gestation

Figure 1 Results of PCs.
Figure 2 Outcomes

Reference

1. Organization WH. Preterm birth. https://www.who.int/news-room/fact-sheets/detail/preterm-birth (accessed 2024-06-16 2024).
2. Norman M, Padkaer Petersen J, Stensvold HJ, et al. Preterm birth in the Nordic countries-Capacity, management and outcome in neonatal care. *Acta Paediatr* 2023; **112**(7): 1422-33.
3. Goldenberg RL, Culhane JF, Iams JD, Romero R. Epidemiology and causes of preterm birth. *The lancet* 2008; **371**(9606): 75-84.
4. Sucksdorff M, Lehtonen L, Chudal R, et al. Preterm birth and poor fetal growth as risk factors of attention-deficit/hyperactivity disorder. *Pediatrics* 2015; **136**(3): e599-e608.
5. Hamilton BE, Martin JA, Osterman MJ. Births: preliminary data for 2015. 2016.
6. Loftin RW, Habli M, Snyder CC, Cormier CM, Lewis DF, DeFranco EA. Late preterm birth. *Reviews in obstetrics and gynecology* 2010; **3**(1): 10.
7. Hong T, Xiao-yu Z. Clinical problems in late preterm infants. *J Perinat Med* 2013; **16**(3): 189-91.
8. McIntire D, Leveno K. Neonatal mortality and morbidity rates in late preterm births compared to births at term. *American Journal of Obstetrics & Gynecology* 2006; **195**(6): S221.
9. Baron IS, Erickson K, Ahronovich MD, Baker R, Litman FR. Cognitive deficit in preschoolers born late-preterm. *Early human development* 2011; **87**(2): 115-9.
10. van Baar AL, Vermaas J, Knots E, de Kleine MJ, Soons P. Functioning at school age of moderately preterm children born at 32 to 36 weeks' gestational age. *Pediatrics* 2009; **124**(1): 251-7.
11. Morse SB, Zheng H, Tang Y, Roth J. Early school-age outcomes of late preterm infants. *Pediatrics* 2009; **123**(4): e622-e9.
12. Cheong JL, Doyle LW, Burnett AC, et al. Association Between Moderate and Late Preterm Birth and Neurodevelopment and Social-Emotional Development at Age 2 Years. *JAMA Pediatrics* 2017; **171**(4): e164805-e.
13. Reuner G, Hassenpflug A, Pietz J, Philippi H. Long-term development of low-risk low birth weight preterm born infants: neurodevelopmental aspects from childhood to late adolescence. *Early human development* 2009; **85**(7): 409-13.
14. Woythaler MA, McCormick MC, Smith VC. Late preterm infants have worse 24-month neurodevelopmental outcomes than term infants. *Pediatrics* 2011; **127**(3): e622-e9.
15. Voigt B, Pietz J, Pauen S, Kliegel M, Reuner G. Cognitive development in very vs. moderately to late preterm and full-term children: Can effortful control account for group differences in toddlerhood? *Early Human Development* 2012; **88**(5): 307-13.
16. Lee HJ, Park H-K. Neurodevelopmental outcome of preterm infants at childhood: cognition and language. *Hanyang Medical Reviews* 2016; **36**(1).
17. Chan E, Quigley MA. School performance at age 7 years in late preterm and early term birth: a cohort study. *Arch Dis Child Fetal Neonatal Ed* 2014; **99**(6): F451-7.
18. Gurka MJ, LoCasale-Crouch J, Blackman JA. Long-term cognition, achievement, socioemotional, and behavioral development of healthy late-preterm infants. *Archives of pediatrics & adolescent medicine* 2010; **164**(6): 525-32.
19. Odd DE, Emond A, Whitelaw A. Long-term cognitive outcomes of infants born moderately and late preterm. *Dev Med Child Neurol* 2012; **54**(8): 704-9.
20. Mollon J, Knowles EEM, Mathias SR, et al. Genetic influence on cognitive development between childhood and adulthood. *Molecular Psychiatry* 2021; **26**(2): 656-65.
21. D'Onofrio BM, Class QA, Rickert ME, Larsson H, Långström N, Lichtenstein P. Preterm birth and mortality and morbidity: a population-based quasi-experimental study. *JAMA Psychiatry* 2013; **70**(11): 1231-40.
22. Yin W, Döring N, Persson MSM, et al. Gestational age and risk of intellectual disability: a population-based cohort study. *Arch Dis Child* 2022; **107**(9): 826-32.
23. Linsell L, Johnson S, Wolke D, et al. Cognitive trajectories from infancy to early adulthood following birth before 26 weeks of gestation: a prospective, population-based cohort study. *Arch Dis Child* 2018; **103**(4): 363-70.

24. Luttikhuizen dos Santos ES, de Kieviet JF, Königs M, van Elburg RM, Oosterlaan J. Predictive value of the Bayley scales of infant development on development of very preterm/very low birth weight children: a meta-analysis. *Early Hum Dev* 2013; **89**(7): 487-96.
25. Rose SA, Feldman JF, Jankowski JJ, Van Rossem R. Basic information processing abilities at 11 years account for deficits in IQ associated with preterm birth. *Intelligence* 2011; **39**(4): 198-209.
26. Böhm B, Katz-Salamon M, Smedler A-C, Lagercrantz H, Forssberg H. Developmental risks and protective factors for influencing cognitive outcome at 5½ years of age in very-low-birthweight children. *Developmental Medicine and Child Neurology* 2002; **44**(8): 508-16.
27. Murray AL, Scratch SE, Thompson DK, et al. Neonatal brain pathology predicts adverse attention and processing speed outcomes in very preterm and/or very low birth weight children. *Neuropsychology* 2014; **28**(4): 552.
28. Romero R, Dey SK, Fisher SJ. Preterm labor: one syndrome, many causes. *Science* 2014; **345**(6198): 760-5.
29. Casey BJ, Cannonier T, Conley MI, et al. The Adolescent Brain Cognitive Development (ABCD) study: Imaging acquisition across 21 sites. *Dev Cogn Neurosci* 2018; **32**: 43-54.
30. Feldstein Ewing SW, Chang L, Cottler LB, Tapert SF, Dowling GJ, Brown SA. Approaching Retention within the ABCD Study. *Dev Cogn Neurosci* 2018; **32**: 130-7.
31. Karcher NR, Barch DM. The ABCD study: understanding the development of risk for mental and physical health outcomes. *Neuropsychopharmacology* 2021; **46**(1): 131-42.
32. Hagler Jr DJ, Hatton S, Cornejo MD, et al. Image processing and analysis methods for the Adolescent Brain Cognitive Development Study. *Neuroimage* 2019; **202**: 116091.
33. Uban KA, Horton MK, Jacobus J, et al. Biospecimens and the ABCD study: Rationale, methods of collection, measurement and early data. *Dev Cogn Neurosci* 2018; **32**: 97-106.
34. Zucker RA, Gonzalez R, Feldstein Ewing SW, et al. Assessment of culture and environment in the Adolescent Brain and Cognitive Development Study: Rationale, description of measures, and early data. *Dev Cogn Neurosci* 2018; **32**: 107-20.
35. Barch DM, Albaugh MD, Avenevoli S, et al. Demographic, physical and mental health assessments in the adolescent brain and cognitive development study: Rationale and description. *Dev Cogn Neurosci* 2018; **32**: 55-66.
36. Luciana M, Bjork JM, Nagel BJ, et al. Adolescent neurocognitive development and impacts of substance use: Overview of the adolescent brain cognitive development (ABCD) baseline neurocognition battery. *Dev Cogn Neurosci* 2018; **32**: 67-79.
37. Kind AJH, Jencks S, Brock J, et al. Neighborhood socioeconomic disadvantage and 30-day rehospitalization: a retrospective cohort study. *Annals of internal medicine* 2014; **161**(11): 765-74.
38. Baurley JW, Edlund CK, Pardamean CI, Conti DV, Bergen AW. Smokescreen: a targeted genotyping array for addiction research. *BMC genomics* 2016; **17**(128): 145-.
39. Corresponding GPC. An integrated map of genetic variation from 1,092 human genomes. *Nature* 2012; **491**(7422): 56-65.
40. Howie B, Donnelly P, Marchini J. 1,000 Genomes haplotypes—Phase 3 integrated variant set release in NCBI build 37 (hg19) coordinates. 2015.
41. Lam M, Awasthi S, Watson HJ, et al. RICOPILI: Rapid Imputation for COnsortias PIpeLIne. *Bioinformatics* 2020; **36**(3): 930-3.
42. Nielsen TT, Duan J, Levey DF, et al. Disentangling the shared genetics of ADHD, cannabis use disorder and cannabis use and prediction of cannabis use disorder in ADHD. *medRxiv* 2024: 2024.02. 22.24303124.
43. Zhou H, Kember RL, Deak JD, et al. Multi-ancestry study of the genetics of problematic alcohol use in over 1 million individuals. *Nature Medicine* 2023; **29**(12): 3184-92.
44. Choi SW, O'Reilly PF. PRSice-2: Polygenic Risk Score software for biobank-scale data. *Gigascience* 2019; **8**(7).
45. Lee JJ, Wedow R, Okbay A, et al. Gene discovery and polygenic prediction from a genome-wide association study of educational attainment in 1.1 million individuals. *Nature genetics* 2018; **50**(8): 1112-21.
46. Turley P, Walters RK, Maghzian O, et al. Multi-trait analysis of genome-wide association summary statistics using MTAG. *Nature genetics* 2018; **50**(2): 229-37.

Appendix

Socioeconomic status

SES was defined as the first principal component from a probabilistic principal component analysis (PCA), capturing 65% of the variance in total household income, highest parental education, and neighbourhood quality. Children missing more than one of these SES measures were excluded. Household income was determined by the combined annual income of all family members over the past 12 months, categorized as less than $49,999 (1), $50,000–74,999 (2), $75,000–99,999 (3); $100,000–199,999 (4); and greater than $200,000 (5). Parental education was categorized into middle school or less (1), some high school (2), high school graduate (3), some college/associate degree (4), bachelor's degree (5), master's degree (6), or professional degree (7). The neighbourhood quality was determined using the area deprivation index, calculated from the American Community Survey using the address of the primary residency.[37] The SES composite and each subcomponent were normalized (mean=0, SD=1).

Polygenic Score Derivation and Analyses

Genotyping, quality control, and imputation

Saliva samples were collected from all the children during the T_0 visit and genotyped using Rutgers University Cell and DNA repository using the Smokescreen array consisting of 646,247 genetic variants.[38]

Quality control, imputation, and genetic PCA were performed by the National Bioinformatics Infrastructure Sweden (NBIS). The following pre-processing steps were conducted. Briefly, single nucleotide polymorphisms (SNPs) with call rates < 98% or minor allele frequencies (MAFs) < 1% were excluded before imputation. Individuals with high rates of missingness (> 2%) and absolute autosomal heterozygosity > 0.2 were excluded, resulting in 10,069 children and 430,622 genetic variants. Haplotypes were prephased using SHAPEIT2, and genetic markers were imputed using IMPUTE4 software.

We utilized the 1000 Genomes haplotypes—Phase 3 integrated variant set release in NCBI build 37 (hg19) coordinates as reference populations. This dataset consists of 2504 samples and 5008 haplotypes from Europeans, Africans, East Asians, Southern Asians, and Americans (https://mathgen.stats.ox.ac.uk/impute/1000GP_Phase3.html). We used this imputation since it

provides better concordance in diverse human populations.[39,40] After that, genotypes with an INFO score < 0.3 or MAF < 0.001% were excluded, which yielded 40,637,119 SNPs in a total of 10,069 children.

The PCA module, as implemented in RICOPILI,[41] was used to check for outliers and control population structure. SNPs were pruned so that there was little linkage disequilibrium (LD) between SNPs (R2 < 0.2, 200 SNP window: Plink–indep-pairwise 200 100 0.2). LD pruning was repeated until 100 K SNPs were reached. The resulting SNPs were then projected into the PCA.[42,43] We utilized the first 20 principal components (20PCs) from the genetic PCA.

cogPGS calculation

We created polygenic scores for cognitive performance (cogPGS) in each child using PRSice-2,[44] which involved summing the effect sizes of thousands of SNPs (weighted by the presence of effect alleles in each child). These SNPs were discovered by large genome-wide association studies (GWAS) on educational attainment, mathematical ability, and general cognitive ability.[45] Details regarding the effect sizes and p values of their SNPs can be assessed through the Social Science Genetics Association Consortium (https://www.thessgac.org/data).

We utilized the data provided by the consortium from a multitrait analysis of GWAS,[46] which, in our case, represents a joint polygenic score focused on a GWAS of cognitive performance and complemented by information from a GWAS on educational attainment, a GWAS on the highest-level math class completed, and a GWAS on self-reported math ability. This joint analysis is ideal because pairwise genetic correlations of these traits were high,[45] and these GWAS had hundreds of thousands of individuals. Such a large sample size allows new studies to detect effects in samples of a few hundred individuals with 80% statistical power.

To construct the cogPGS, we performed clumping and pruning to remove nearby SNPs that are correlated with each other. The clumping sliding window was 250 kb, with the linkage disequilibrium clumping set to $r^2 > 0.25$. We included the weightings of all SNPs, regardless of their p-value from the GWAS (p = 1.00 threshold), resulting in 5255 SNPs. Finally, we normalized (mean=0, SD=1) the cogPGS to fairly compare their effects on different phenotypes. For the present study, we used cogPGS to reflect the genetic predisposition of cognitive performance and included 20 genetic principal components (PCs) to account for the possibility of population stratification within the Add Health European-ancestry subsample in the same model.
