# Supplementary material for: Gestational Age and Cognitive Development in Childhood
Source: JAMA Netw Open. 2025 Apr 14;8(4):e254580. doi: 10.1001/jamanetworkopen.2025.4580 (PMC11997729; doi:10.1001/jamanetworkopen.2025.4580)
Supplement: Supplement 3. — Data Sharing Statement [file jamanetwopen-e254580-s003.pdf]

# Data Sharing Statement

Nivins. Gestational Age and Cognitive Development in Childhood. *JAMA Netw Open*. Published April 14, 2025. doi:10.1001/jamanetworkopen.2025.4580

**Data**
**Data available:** Yes
**Data types:** Deidentified participant data
**How to access data:** https://nda.nih.gov/abcd
**When available:** With publication

**Supporting Documents**
**Document types:** Statistical/analytic code
**How to access documents:** samson.nivins@ki.se
**When available:** With publication

**Additional Information**
**Who can access the data:** researchers whose proposed use of the data has been approved
**Types of analyses:** for any research purpose
**Mechanisms of data availability:** after signed data access agreement
